# Distinct infant resistome trajectories shaped by country income and geography revealed through global metagenomics reanalysis

**DOI:** 10.1038/s44259-026-00194-8

**Published:** 2026-04-15

**Authors:** Charlie C. Luchen, Gonçalo J. Piedade, Mwelwa Chibuye, Michelo Simuyandi, Caroline C. Chisenga, Roma Chilengi, Samuel Bosomprah, Constance Schultsz, Daniel R. Mende, Vanessa C. Harris

**Affiliations:** 1 Department of Global Health, Amsterdam Institute for Global Health and Development, Amsterdam UMC, University of Amsterdam, Amsterdam, The Netherlands; 2 Research Division, Centre for Infectious Disease Research in Zambia, Lusaka, Zambia; 3 Zambia National Public Health Institute, Ministry of Health, Lusaka, Zambia; 4 Republic of Zambia State House, Lusaka, Zambia; 5 Department of Biostatistics, School of Public Health, University of Ghana, Accra, Ghana; 6 Department of Medical Microbiology, Amsterdam UMC, location University of Amsterdam, Amsterdam, The Netherlands; 7 Amsterdam Institute for Immunology and Infectious diseases, Amsterdam University Medical Center, Amsterdam, The Netherlands; 8 Human Biology Microbiome Quantum Research Center (WPI-Bio2Q), Keio University, Tokyo, Japan; 9 Department of Internal Medicine, Division of Infectious Diseases, Amsterdam UMC, University of Amsterdam, Amsterdam, The Netherlands

**Keywords:** Computational biology and bioinformatics, Diseases, Gastroenterology, Health care, Medical research, Microbiology

## Abstract

Antimicrobial resistance (AMR) costs lives, diminishes antimicrobial effectiveness and increases health care costs. We conducted a re-analysis of pooled fecal metagenomes from individual participants to characterise AMR gene (ARG) distributions in 0–2 year-old healthy infants across income and geography. From 2275 screened studies, we included nine datasets and 1944 fecal metagenomes. Resistome gene identifier (RGI) was used to identify ARGs, and gut microbiomes were profiled using Sylph. We assessed associations between ARGs, *Escherichia coli* abundance, and national-level indicators. In the first 3 months of life, ARG abundance patterns were not significantly different across income groups; however, by 6 months of age, infants in LICs had higher ARG abundance, associated with increased *E. coli* carriage. Caesarean section rates, antibiotic use, and income inequality positively correlated with ARG abundance in younger infants; physician density negatively correlated with ARG abundance in older children. These descriptive age- and context-specific associations may inform interventions to mitigate the carriage and spread of ARGs and the rise of AMR in vulnerable pediatric populations.

## Introduction

Antimicrobial resistance (AMR), defined as the ability of microorganisms to survive or grow despite the presence of an antimicrobial, threatens human health worldwide. This phenomenon makes infections more challenging to treat, leading to longer illness duration and increased health care costs^1^. The gut microbiome is a reservoir for antimicrobial resistance genes (ARGs) and can facilitate the horizontal gene transfer of ARGs between commensal organisms and pathogens within individuals^2^. Therefore, characterising ARGs in the microbiome, collectively described as the resistome, is useful for understanding the potential for human pathogens to acquire ARGs and risk of spread of microorganisms with AMR from individuals to environments and communities^3^.

Children under 2 years of age in low- and middle-income settings are at high risk for infections with AMR pathogens^4^, requiring an understanding of factors shaping their resistome. Infants have an immature immune system, a high incidence of infectious diseases, and an increased risk for severe infectious disease^3,5^. Higher incidences of infectious diseases, coupled with limited diagnostic and laboratory capacity results in multiple exposures to appropriate and inappropriate antibiotics. For instance, children in LMICs receive an average of 11 courses of antibiotics in the first 2 years of life, peaking around 11 months of age^6^, as compared to only one to two courses in the first year of life in high-income countries (HICs)^7^. These factors are associated with ARG expansion in children’s intestinal microbiomes^8,9^. While studies have extensively described the risk factors for ARG expansion in the microbiota of HIC children, much less is known about risks in children residing in low and middle-income countries with the highest risk of disease from AMR-bearing organisms^3,10^.

Alongside infectious disease incidence and antibiotic exposures, risk factors related to fecal and antibiotic environmental exposures and socioeconomic status (SES) are likely important determinants of resistome composition and AMR risk in children across geographical settings that are insufficiently accounted for in single-country studies^11^. Regional variations in AMR patterns at individual and population levels have been associated with differences in environmental and SES factors^4^. A recent modelling study highlights that country-level variables, such as water, sanitation, and hygiene (WASH), are associated with global variations in ARG distribution in adults^12^. Our study investigates these patterns specifically in children, assessing regional variations in children’s ARG gene carriage to identify environmental and SES risk factors and inform regionally relevant interventions.

Microbiome composition is also closely linked to ARG carriage. Differences in microbiome taxonomy across ages and geographic regions have been extensively investigated in children^13–15^. However, while this taxonomic data has provided valuable insights, research into how these taxonomic differences influence the resistome, particularly in early life, remains limited^8^. Fewer studies have investigated the interplay between microbiome composition and ARGs, including how these relationships vary across regions and SES contexts or change temporally during critical stages of infant development^11^. Understanding factors associated with ARG carriage at a global scale is particularly challenging due to the lack of data from low-income settings and a predominance of studies from high-income regions focusing on infants with disease in clinical settings.

In this study, we address this research gap by re-analysing publicly available participant-level shotgun metagenomic sequencing data using a single pipeline to compare the resistome of healthy children under the age of two across diverse geographical and SES settings. Through this comparison, we aim to identify key factors associated with ARG carriage variation and inform interventions that reduce the risk of infections with AMR bacteria in early life.

## Results

**Fig. 1:**
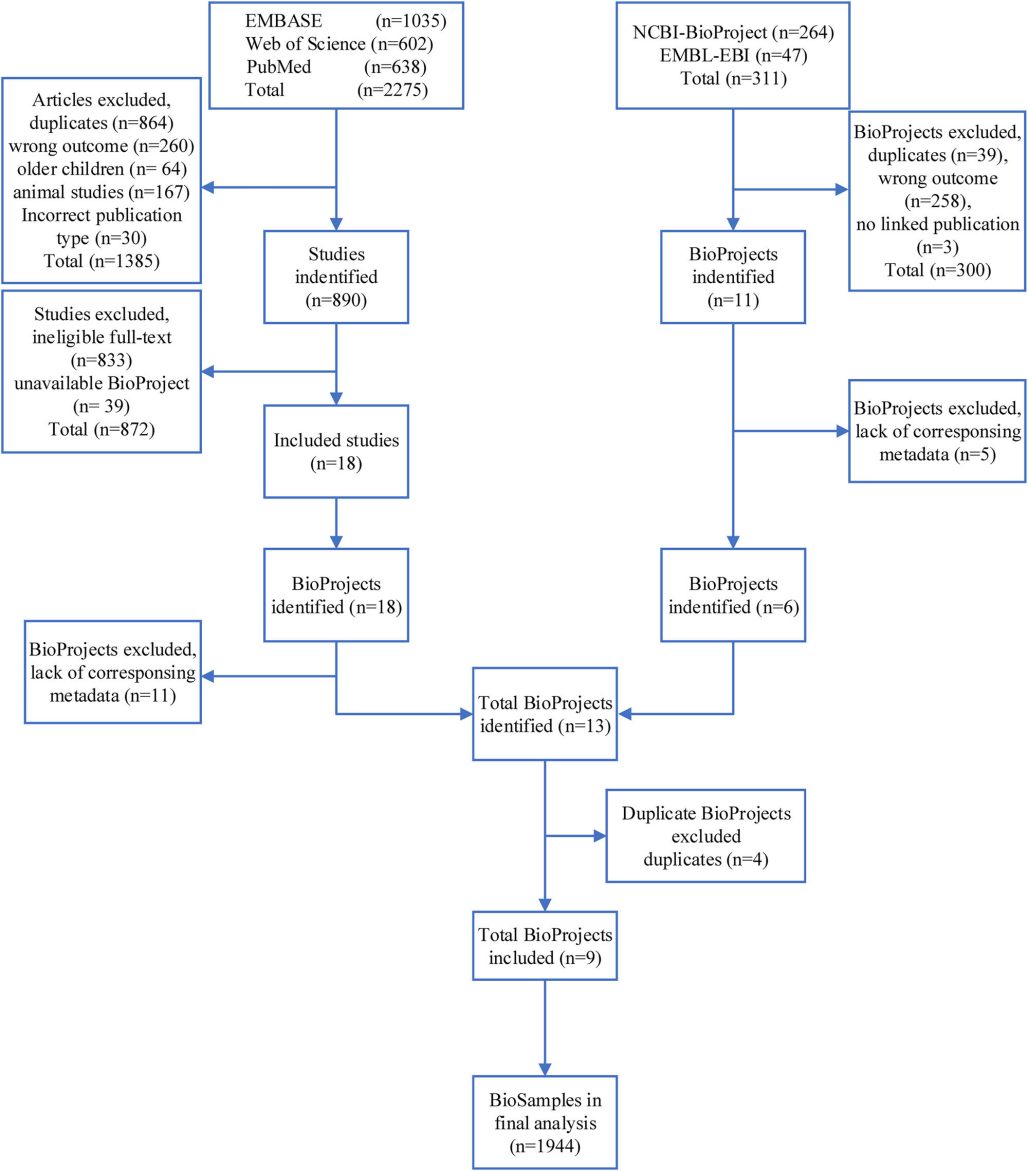
Flowchart of the included BioSamples. *n* number

Our systematic search strategy identified 2275 articles. We screened these based on title and abstract, excluding 1385 articles due to duplicates and failure to meet the inclusion criteria. Following full-text screening of the remaining 890 articles, 872 were excluded due to being ineligible for full-text (*n* = 833) or the unavailability of a corresponding BioProjects (*n* = 39), leaving 18 relevant articles corresponding to 18 BioProjects. We identified a total of 311 BioProjects during the complementary database search. After detailed screening, 300 BioProjects were excluded due to incorrect outcomes, such as reporting solely on 16S RNA, lack of linked publication, or duplication, leaving 11 BioProjects. After excluding BioProjects that lacked metadata, we merged 13 BioProjects identified by both search strategies. Following duplicate removal, nine unique BioProjects, comprising 1944 BioSamples, were included in the final analysis (Fig. 1, and Table 1).

**Table 1:** Characteristics of the included BioSamples

Income group	Country	BioProject	Author (Year)	Mean age, months (SD)	BioSamples (*N*)	Age group, months (*n*)			
						<3	3–6	6–12	>12
Low				12.4 (4.9)	105	3	2	53	47
	Ethiopia	PRJNA504891^50^	Tett (2019)	10.9 (6.4)	14 (NA)	3	0	5	6
	Mozambique	PRJNA747761^51^	Kim (2022)	12.6 (4.7)	91ʸ	0	2	48	41
Lower middle				6.7 (6.4)	319	105	71	39	104
	Zimbabwe	PRJEB51728^64^	Robertson (2023)	6.7 (6.4)	319 (157)	105	71	39	104
Upper middle				12.6 (5.5)	104	0	7	40	57
	Russia	PRJNA290380^65^	Vatanen (2016)	12.6 (5.5)	104 (47)	0	7	40	57
High				2.4 (3.5)	1416	1104	122	108	76
	Estonia	PRJNA290380^65^	Vatanen (2016)	9.7 (6.5)	76 (48)	11	6	13	46
	Finland	PRJNA290380^65^	Vatanen (2016)	7.6 (5.2)	67 (42)	10	12	18	27
	Germany	PRJEB24041^66^	Korpela (2018)	3.7 (2.7)	11 (NA)	5	3	3	0
	Italy	PRJNA339914^67^	Asnicar (2017)	5.3 (3.5)	8 (5)	5	0	2	1
	Luxembourg	PRJEB24041^66^	Korpela (2018)	9.8 (3.3)	8 (NA)	0	0	6	2
	Sweden	PRJEB6456^14^	Backhed (2015)	5 (4.4)	202 (71)	64	72	66	0
	UK	PRJEB32631^16^	Shao (2019)	0.4 (0.2)	918 (451)	918	0	0	0
	USA	PRJNA633576^68^	Casaburi (2021)	5.9 (0)	126ʸ	94	32	0	0

*SD* standard deviation, *NA* not available, *N* number of participants, ʸ number of participants is the same as the number of BioSamples.

### Baseline characteristics of BioSamples

From the 1944 analysed BioSamples, the United Kingdom (UK) contributed the highest number (*n* = 918, 47.2%)^16^. BioSample representation across income status was skewed towards high-income settings: samples were available from two low-income countries (LICs), one lower-middle-income country (LMIC) and one upper-middle-income country (UMIC), and eight HICs (Table 1). We stratified age into four groups (<3 months, 3–6 months, 6–12 months, and >12 months) to reflect key stages in infant microbiome development influenced by factors such as breastfeeding and dietary transitions. BioSamples from children younger than 3 months (<3 age group) were predominantly from HICs, comprising 91.09% (1104/1212), while only 0.25% (3/1212) were from LICs and 8.66% (105/1212) from LMICs. In contrast, BioSamples for the oldest age quartile (>12 months age group) were well represented in LICs and UMICs, with 16.55% (47/284) and 20.07% (57/284) BioSamples, respectively. The mean age by income status was 12.4 months (SD 4.9) for LICs, 6.7 months (SD 6.4) for LMICs, 12.6 months (SD 5.5) for UMICs, and 2.4 months (SD 3.5) for HICs (Table 1).

### Global infant gut taxonomy and resistome vary by income group and age

**Fig. 2:**
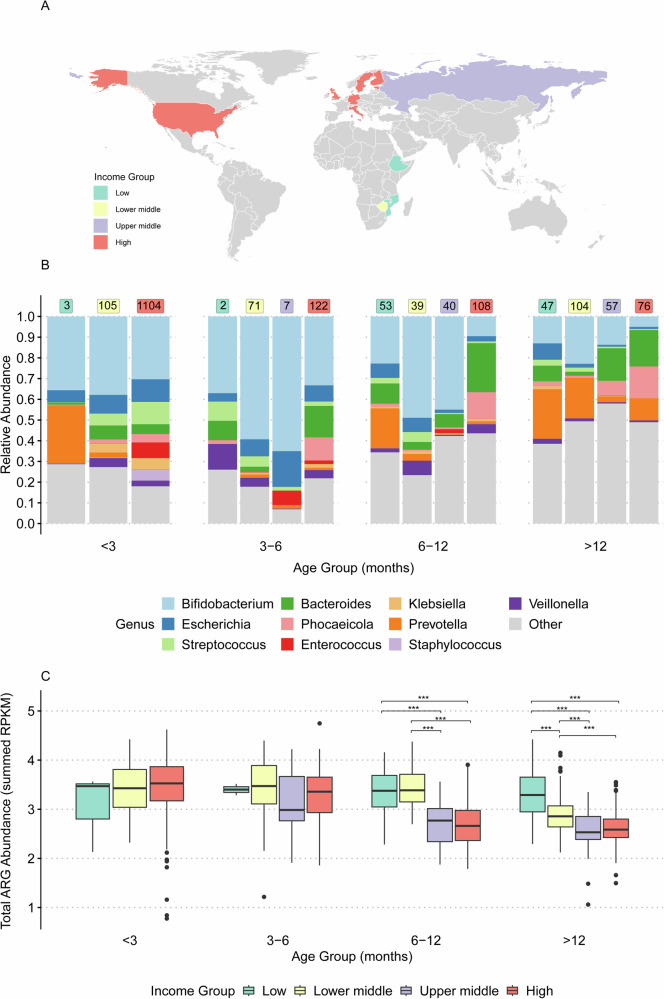
Antimicrobial resistance gene abundance and taxonomy differ by age and country-level income status. **A** Global distribution of origin of BioSamples and coloured by the associated income group categorisation. **B** Taxonomy relative abundance. The bars show the top 10 most abundant genera by age and income group. The text annotations on each bar represent the number of BioSamples profiled per income group (green = LICs, yellow = LMICs, purple = UMICs, and red = HICs). These sample sizes correspond to the groups shown in **C**. **C** ARG abundance showing the association of log 10 transformed RPKM values by age per income grouping. ^***^(*p* < 0.001) indicate pairs that differed significantly using Wilcoxon’s test followed by Holm adjustment. The box plot displays 25th, 50th (median), and 75th percentiles.

The analysed samples spanned 12 countries across four income categories (Fig. 2A), with representation from two LICs (Ethiopia and Mozambique), one LMIC (Zimbabwe), one UMIC (Russia), and eight HICs (Estonia, Finland, Germany, Italy, Luxembourg, Sweden, UK, and USA). We first evaluated differences in bacterial taxonomic composition stratified by income and age groups. Analysis of the top ten most abundant genera revealed that across all income groups, *Bifidobacterium* was consistently more abundant in younger age groups (<3, 3–6, and 6–12 months) and showed a marked decline in the >12-month age group. A similar trend was observed for *Escherichia*, where it generally declined with age; however, in LICs, it increased with age and was significantly more abundant compared to other income groups within the >12 months age group (Dunn’s test, *p* < 0.001) (Supplementary Fig. 2).

In the > 12 months group, *Prevotella* abundance in LICs was significantly higher than in LMICs (Dunn’s test, *p* < 0.01), UMICs (Dunn’s test, *p* < 0.001), and HICs (Dunn’s test, *p* < 0.001) (Fig. 2B, and Supplementary Fig. 2). *Prevotella copri_A* and an unspeciated *Prevotella sp934191715* mainly drove the observed age and income association in *Prevotella* abundance (Supplementary Figs. 2 and 3). BioSamples from LIC under 3 months of age were limited (*n* = 3 Ethiopian infants). Given these limitations, these exploratory analyses suggested that Prevotella relative abundance also differed by age and income group, being significantly higher in LICs samples, compared to LMICs (Dunn’s test, *p* < 0.001) and HICs (Dunn’s test, *p* < 0.001) (Supplementary Fig. 2).

Next, we evaluated trends in total ARG abundance stratified by income and age groups. Generally, ARG abundance decreased as age increased across the income groups, except in LICs, where ARG abundance remained persistently high across the age groups (Fig. 2C). For children under 3 months old, ARG abundance did not differ significantly by income group. This contrasted in older 6–12 and >12-month age groups where ARG abundance was significantly higher (Wilcoxon’s test, *p* < 0.001) in LICs compared to UMICs and HICs (Fig. 2C). To ensure that our results were not an artifact of the abundance metric, we also assessed ARG richness (unique gene count) and Shannon/Simpson diversity (Supplementary Fig. 4). In infants under 3 months, LMIC settings exhibited significantly higher ARG alpha diversity compared to infants from HICs (Wilcoxon’s test, *p* < 0.001) (Supplementary Fig. 4). From 6 months onward, infants from LIC and LMIC countries exhibited significantly higher ARG richness, Shannon diversity, and inverse Simpson indices compared to high-income settings (Wilcoxon’s test, *p* < 0.001) for most comparisons. Notably, this divergence intensified with age, with infants older than 12 months in LICs displaying the highest ARG diversity.

The higher ARG abundance in LICs compared to HICs was mainly driven by a greater abundance of genes encoding resistance against aminoglycosides, lincosamides, macrolides, and fluoroquinolones within the 6–12 months age group, and fluoroquinolones within the >12-month age group (Wilcoxon’s test, *p* < 0.05) (Supplementary Fig. 5).

Feeding method and mode of delivery were significantly associated with ARG abundance. Infants delivered via caesarean section had significantly higher ARG abundance compared to those delivered vaginally (Wilcoxon’s test, *p* < 0.05) (Supplementary Fig. 6A). Differences in ARG abundance were also observed between breastfeeding and formula feeding, as well as between breastfeeding and mixed feeding (Wilcoxon’s test, *p* < 0.05) (Supplementary Fig. 6B). However, these findings should be interpreted with caution due to missing data on delivery modes and feeding practices for several BioSamples (Supplementary Fig. 6C, D). BioSamples with missing metadata are displayed as ‘NA’ for transparency, but were not included in statistical comparisons (Supplementary Fig. 6).

### Differences in ARG distribution are independently influenced by age, income, feeding method, delivery mode

**Fig. 3:**
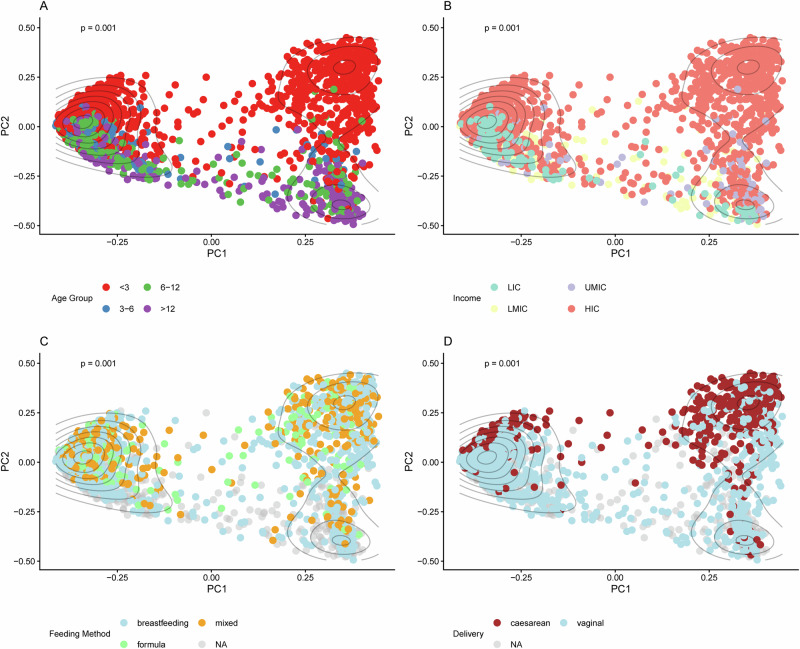
Beta-diversity of ARGs. Across (**A**) age groups, **B** income categories, **C** feeding type and **D** delivery mode. Dots represent individual samples coloured according to the specific variables. PERMANOVA analysis demonstrated significant differences in ARG beta-diversity across all variables (*p*-value = 0.001). NA indicates samples with missing metadata for the respective variable. Black curved lines represent density contours.

To explore further determinants of resistome composition, we assessed differences in ARG beta-diversity across four variables: income category, age, feeding method, and delivery mode (Fig. 3). We observed significant differences in ARG diversity across each of these variables (PERMANOVA, *p* = 0.001 for all analyses). These findings suggest that age, national income level, feeding type, and delivery mode contribute to resistome beta diversity. However, overlapping patterns across the assessed variables highlight potential confounding effects.

### Clustering of ARG abundance reveals distinct high-ARG, moderate-ARG, and low-ARG resistome profiles associated with *E. coli* abundance

In evaluating the ARG abundance of all the samples, we observed a striking separation across PC1 in the principal coordinate analysis (PCoA) ordination (Fig. 3). To formally assess cluster structure, we performed *k*-means clustering and evaluated the optimal number of clusters using gap statistic and silhouette analysis (Supplementary Fig. 7). The gap statistic identified *k* = 3 as optimal (gap = 0.512) (Supplementary Fig. 7A), while silhouette analysis showed *k* = 3 (average width = 0.688) outperformed *k* = 2. We therefore selected *k* = 3 because of the plateau in gap statistic beyond *k* = 3, indicating no further meaningful structure. We therefore partitioned samples into three clusters (Cluster 1, Cluster 2, and Cluster 3). The three identified clusters demonstrated high robustness. Cluster assignments were highly reproducible across 100 random seed initialisations (adjusted Rand index = 0.959), confirming the three clusters represented stable data structures. To assess sensitivity to distance metrics, we repeated the analysis using Jaccard distance instead of Bray–Curtis dissimilarity; both approaches grouped 99% of samples into the same clusters (Supplementary Fig. 8, and Supplementary Table 3), further validating the robustness of the identified clusters.

**Fig. 4:**
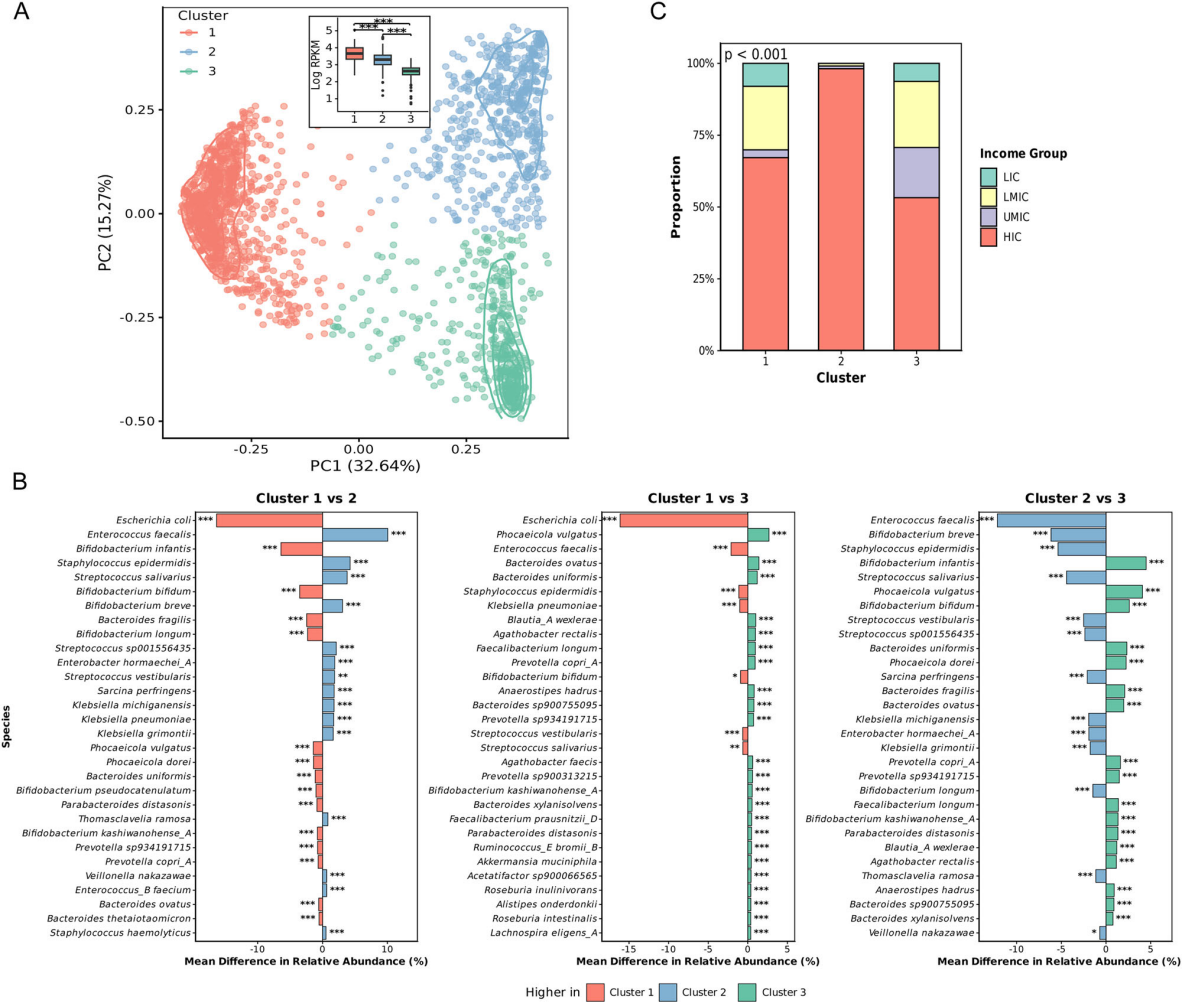
ARGs and taxonomic abundance associate with clustering patterns observed in PCoA. **A** PCoA plot of ARG abundance, coloured according to distinct clusters. The figure boxplot insert shows the distribution of log-transformed (base 10) RPKM values across the three clusters; the red boxplot represents high-ARG cluster, and the blue boxplot represents moderate-ARG cluster while the green boxplot represents the low-ARG cluster. Asterisk (***) indicate statistically significant differences between the clusters. Curved contour lines represent density estimates for each cluster. **B** Mean relative abundance difference between clusters for the top 30 taxa. Holm adjusted *p*-values are displayed as ^*^p < 0.05, ^**^p < 0.01, and ^***^p < 0.001. **C** Proportion of samples from each income group within the three clusters. *P*-value computed using Fisher’s exact test.

Cluster 1 exhibited significantly higher ARG abundance compared to Clusters 2 and 3 (Wilcoxon’s test, *p* < 0.001; Fig. 4A, inset), followed by moderate abundance in Cluster 2, and lowest in Cluster 3. We therefore refer to these as the high-ARG cluster (Cluster 1), moderate-ARG cluster (Cluster 2), and low-ARG cluster (Cluster 3). Taxonomically, the high-ARG cluster was characterised by significantly higher relative abundance of *Escherichia coli* compared to the moderate- and low-ARG clusters (Fig. 3B), while the moderate-ARG cluster was enriched for *Enterococcus faecalis* and *Staphylococcus epidermidis*. Conversely, the low-ARG cluster was dominated by *Phocaeicola vulgatus* (formerly *Bacteroides vulgatus*) and *Bifidobacterium infantis*. These patterns indicate that resistome variation is strongly associated with opportunistic pathogen abundance, particularly Gram-negative Enterobacteriaceae, while health-associated commensal bacteria correspond to lower ARG burden. Furthermore, the three clusters differed significantly by country income level (Fisher’s exact test, *p* < 0.001; Fig. 4C). The high-ARG cluster was enriched for samples from LICs, the moderate-ARG cluster for HICs, and the low-ARG cluster showed more balanced representation across LMICs and UMICs. To further explore taxonomic differences between the clusters, we examined the distribution of *E. coli* abundance along PC1. We found a strong inverse correlation between PC1 scores and *E. coli* abundance (Spearman’s *ρ* = −0.88, *p* < 0.0001; Supplementary Fig. 9).

Beyond *E. coli*, we assessed whether broader microbiome composition predicted resistome structure using Procrustes analysis by comparing PCoA ordinations of taxonomic and ARG abundance. Overall, microbiome and resistome structures showed strong concordance across our dataset (*r* = 0.805, *M*² = 0.352, *p* < 0.001; Supplementary Fig. 10A). Income-stratified analyses revealed that concordance was highest in HICs (*r* = 0.831, *M*² = 0.309, *p* < 0.001, *n* = 1410), followed by UMICs (*r* = 0.672, *M*² = 0.548, *p* < 0.001, *n* = 104), LMICs (*r* = 0.669, *M*² = 0.552, *p* < 0.001, *n* = 319), and LICs (*r* = 0.681, *M*² = 0.536, *p* < 0.001, *n* = 105) (Supplementary Fig. 10B–E). These findings indicated that microbiome composition is strongly concordant with resistome structure across all income settings, with the strongest relationship observed in HICs. Having established that microbiome composition associates with resistome structure, we next identified specific taxa associated with ARG abundance using Spearman correlations across all bacterial species and ARG classes (Supplementary Fig. 11), which revealed significant positive associations between *E. coli* abundance and ARG abundance across multiple antibiotic classes. Specifically, *E. coli* abundance was positively correlated with ARG abundance for aminoglycosides (*r* = 0.5), beta-lactams (*r* = 0.4), sulfonamides (*r* = 0.4), and fluoroquinolones (*r* = 0.4), but negatively correlated with streptogramins (*r* = −0.1) (Supplementary Fig. 11). Therefore, despite samples being derived from multiple geographic settings, we observed three major clusters of resistome composition in our dataset. These clusters were primarily distinguished by RPKM values, which represent ARG abundance, and large differences in *E. coli* abundance, with increased *E. coli* abundance associating with increased ARG abundance against multiple drug classes.

### Association of national-level factors with resistome and *Escherichia coli* abundance

Lastly, we were interested in understanding whether national-level variables, representing varying risks for faecal exposures, infectious diseases, and antibiotic exposures, and health care utilisation, were associated with both ARG and *E. coli* abundance. We hypothesised that these factors may mediate the abundance of both outcomes. To test this, we used a lasso regression model with cross-fitting partialling-out to examine the associations between ARG and *E. coli* abundance and the following factors: the defined daily dose of antibiotics (DDD) consumed, the number of medical doctors per 10,000 population, the Gini index, and C-section rates (Supplementary Fig. 12A, B). Given our earlier finding of the importance of age in shaping the microbiome and resistome, we included age as a variable in the main model. Also, we ran a separate, age-stratified model to explore potential age-related differences.

**Fig. 5:**
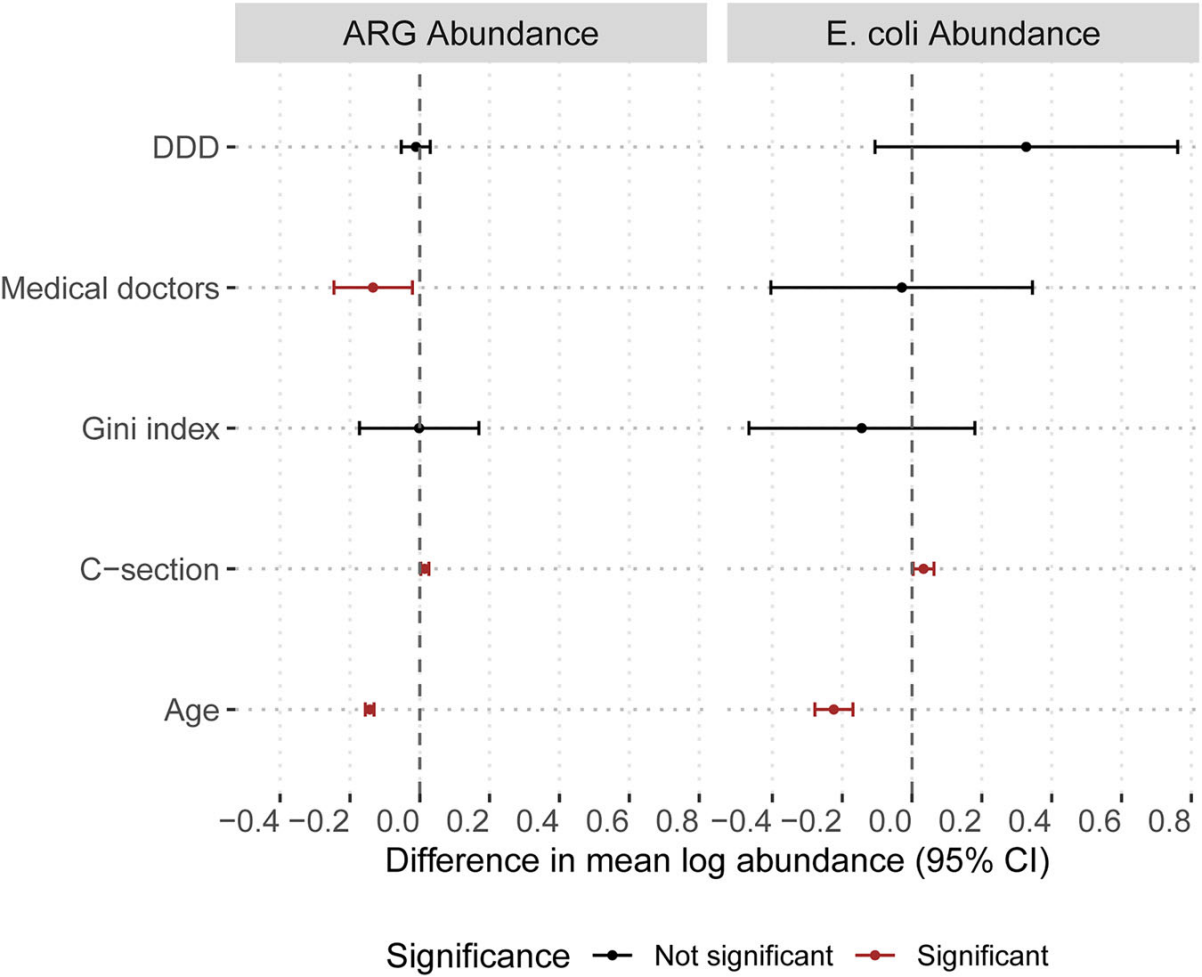
ARG and *E. coli* abundance are associated with age and national-level variables. Each dot represents the difference in mean log-transformed ARG or *E. coli* abundance per standard deviation increase in each predictor, and horizontal lines represent the 95% upper and lower confidence intervals. The variables were standardised by subtracting their mean and dividing by their standard deviation (z-score standardisation) before fitting the model. Significant associations (*p* < 0.05) are shown in red, while non-significant associations are in black. DDD stands for defined daily dose of antibiotics per 1000 population; medical doctors refer to the number of medical doctors per 10,000 population; and C-section denotes caesarean section births per 1000 population.

Across the entire dataset, we observed that the number of medical doctors at the national level was negatively associated with ARG abundance (*β* = −0.134, 95% CI: −0.246 to −0.021, *p* = 0.0202). In contrast, C-section rates were positively associated with ARG (*β* = 0.014, 95% CI: 0.003–0.026, *p* = 0.0132) and *E. coli* abundances (*β* = 0.033, 95% CI: 0.003–0.063, *p* = 0.0001). Age was negatively associated with ARG (β = -0.143, 95% CI: −0.156 to −0.131, *p* < 0.0001) and *E. coli* abundances (*β* = −0.224, 95% CI: −0.278 to −0.169, *p* = 0.032) (Fig. 5, and Supplementary Table 4).

Interestingly, associations between the national-level variables and ARG abundance varied significantly by younger and older groups, with similar patterns observed for *E. coli* abundance (Supplementary Fig. 13, and Supplementary Table 5). Specifically, among children under 3 months of age, higher DDD (*β* = 0.409, 95% CI: 0.069–0.750, *p* = 0.0185), Gini index (*β* = 0.421, 95% CI: 0.149–0.693, *p* = 0.0025), and C-section rates (*β* = 0.052, 95% CI: 0.000–0.103, *p* = 0.0482) were positively associated with ARG abundance. Higher numbers of medical doctors (*β* = 1.717, 95% CI: 0.647–2.787, *p* = 0.0017) and Gini index (*β* = 1.917, 95% CI: 0.079–3.755, *p* = 0.0414) correlated with increased *E. coli* abundance. Within the 3–6-month age group, increases in DDD (*β* = 3.048, 95% CI: 2.169–3.927, *p* < 0.0001), medical doctors (*β* = 1.929, 95% CI: 1.114–2.744, *p* < 0.0001), Gini index (*β* = 3.037, 95% CI: 2.404–3.671, *p* < 0.0001), and C-section rates (*β* = 0.234, 95% CI: 0.098–0.370, *p* = 0.0009) were all positively associated with elevated ARG abundance. *E. coli* abundance in this age group was positively associated only with DDD (*β* = 0.565, 95% CI: 0.144–0.985, *p* = 0.0094).

Patterns differed above 6 months of age. Among children aged 6–12 months, higher medical doctor numbers negatively associated with ARG (β = −0.363, 95% CI: −0.486 to −0.239, *p* < 0.0001) and *E. coli* abundances (*β* = −1.064, 95% CI: −1.291 to −0.837, *p* = 0.0001). Increased C-section rates were associated with higher ARG (*β* = 0.036, 95% CI: 0.023–0.049, *p* < 0.0001) and *E. coli* abundances (*β* = 0.155, 95% CI: 0.093–0.217, *p* = 0.0001). Increased DDD was associated with higher *E. coli* abundance (*β* = 0.881, 95% CI: 0.415–1.346, *p* = 0.0003). For children older than 12 months, only higher numbers of medical doctors were significantly associated with reduced ARG (*β* = −0.478, 95% CI: −0.893 to −0.062, *p* = 0.0251) and *E. coli* abundances (*β* = −1.024, 95% CI: −1.441 to −0.608, *p* = 0.0001) (Supplementary Fig. 13, and Supplementary Table 5).

These results generally indicate that below 6 months of age, higher numbers of medical doctors, C-section rates, high Gini index, and high DDD associate with an increased ARG and *E. coli* abundance. However, from 6 months of age onward, lower number of medical doctors associate with increased ARG and *E. coli* abundance.

## Discussion

This study uses a re-analysis of participant-level metagenomic data to describe the infant resistome by age, geography, and country-level indicators, addressing critical gaps in understanding the global distribution of ARGs in infancy. Importantly, our results identify age- and income-specific patterns in ARG abundance, with *E. coli* abundance strongly correlating with ARG carriage. We observed that at young ages, high defined daily doses of antibiotics and C-section rates alongside SES parameters, such as a high number of medical doctors per 10,000 population and Gini index, are significantly associated with ARG and *E. coli* expansion. However, by 6–12 months of age, these trends do not persist. Given limited LIC representation in infants <3 months (*n* = 3), income comparisons in this age stratum are exploratory. We describe a disproportionate burden of ARGs in children from LICs from 6 to 12 months of age, where likely lower numbers of medical doctors, limited diagnostic resources and restricted access to care may co-occur with higher ARG carriage. Together, these findings outline two possible risk profiles for high ARG and *E. coli* carriage in children, notably <3-month-old infants in higher-resourced settings vs >6-month-old children in low-resource settings.

Distinct taxonomic compositional differences underpinned the observed ARG abundance patterns across age and income. For instance, *Bifidobacteria*, commonly found in younger infants, decreased with age across all income levels, consistent with previous studies^13^. Moreover, *Bifidobacteria* may confer protection against ARG carriage, as species within this genus negatively correlated with ARG abundance across multiple drug classes, aligning with a previous report in neonates^17^. On the other hand, genera such as *Prevotella* were more abundant in younger infants from LICs, reflecting the characteristic microbiomes of these geographic areas^18^. *Escherichia* abundance was significantly higher in older children in LICs and was strongly associated with the observed differences in resistome abundance across income groups as infants aged.

The ability of *E. coli* to harbour diverse ARGs across multiple drug classes is well described in the literature. Commensal *E. coli* functions as a reservoir, harbouring and transmitting resistance determinants via mobile genetic elements^19,20^. *E. coli* can contribute to AMR in enteric pathogens through the horizontal gene transfer of mobile elements, such as plasmids with ARGs^21^. While our cross-sectional design precludes causal inference, the strong correlation between *E. coli* and ARG abundance reflects its intrinsic capacity to carry multiple resistance genes. This association between *E. coli* and ARG carriage was also observed in a study of Danish children aged 1 year^3^. However, *E. coli* is not the sole contributor to infant ARG carriage. Other members of Enterobacteriaceae (including *Klebsiella* and *Enterobacter* spp.) also serve as important ARG reservoirs in infant microbiomes^22,23^, and our focus on *E. coli* reflects its predominance in our dataset rather than exclusivity as an ARG carrier. Complementing this, we found a moderate-ARG cluster enriched in *Enterococcus faecalis* and *S. epidermidis*, both recognised nosocomial pathogens that frequently colonise hospital surfaces and cause device-associated infections in neonatal intensive care units^24,25^. Recent genomic characterisation of *Enterococcus* isolates from healthy European infants revealed that the majority harbour AMR genes, with rich reservoirs of plasmids even in community settings^26^. These opportunistic pathogens persist in healthcare environments despite routine disinfection, serving as reservoirs for AMR transmission.

In resource-limited settings, several exposures are likely to contribute to higher carriage of Enterobacteriaceae and their ARGs, including more frequent antibiotic use, repeated faecal exposure through close contact with livestock, and limited access to safely managed water and sanitation services^27^. As children begin to crawl, walk, and transition to complementary foods, these conditions may increase opportunities for faecal oral transmission of *E. coli* and other Enterobacteriaceae. These exposures may explain the observed persistent ARG elevation in LICs at ages 6–12 months and beyond, whereas HIC infants show declining ARG abundance with age^28,29^. This higher and more sustained ARG burden in LIC infants was associated with an enrichment of aminoglycoside, fluoroquinolone, and macrolide resistance genes in this group. Fluoroquinolones and macrolides are commonly used to treat bacterial diarrhoeal disease in many LMICs, where diarrhoea incidence in infancy remains high due to incomplete vaccine impact^30^ and inadequate sanitation and hygiene infrastructure^31^. Although our cross-sectional design precludes causal inference, we hypothesise that the combination of ongoing enteric infections, antibiotic treatment patterns, and repeated environmental faecal exposures may help to maintain persistent ARG carriage in older infants in LICs, in contrast to the declining ARG burden observed in HIC infants.

We focused on healthy children to establish how baseline resistome patterns are shaped by SES exposures rather than illness or treatment. Understanding this baseline is critical because it defines the pre-existing ARG reservoir from which resistant infections emerge. Metagenomic studies of children with diarrheal diseases report substantially higher ARG abundance and diversity than healthy controls. For example, Pakistani children with diarrheagenic infections harboured 63 ARGs versus 17 in healthy children, with similar patterns observed in Ethiopian cohorts^32,33^. Our observation that healthy LIC children aged 6–12 months already carry an elevated ARG burden suggests their baseline reservoir is elevated before any diarrheal episode. This implies that when these children develop diarrhoea, they may experience greater ARG selection and transmission risk than HIC children with lower baseline carriage, emphasising the importance of preventive interventions targeting environmental and socioeconomic drivers before disease occurs.

Variation in ARG and *E. coli* abundance was associated with national-level physician density. Interestingly, we observed a positive correlation between the density of medical doctors and the abundance of *E. coli* in children under the age of 3 months. While this association may not imply a direct causal relationship, areas with high physician density may be associated with more frequent healthcare visits and greater access to hospital-based delivery during infancy. However, the elevated ARG burden in these settings cannot be attributed to solely *E. coli* expansion, as *E. coli* abundance is typically higher in vaginal deliveries than C-sections^34^. Rather, healthcare-associated exposures likely perturb the broader microbiome composition, selecting for multiple ARG-carrying taxa. Separately, our findings showed a positive association between ARG abundance and rates of C-section births. C-section births are often accompanied with intrapartum antibiotic prophylaxis (IAP) administration during delivery which could select for ARG-carrying bacteria^35,36^. The high prevalence of C-section rates in high-income settings, coupled with IAP, may transiently elevate ARG burden in early HIC infancy. By 6 months of age, however, this healthcare-related effect attenuates as the microbiome matures, unveiling a divergence in resistance patterns. ARG burden declines in HIC children while remaining persistently elevated in older LIC children, where environmental factors such as poor WASH conditions may dominate as children become mobile and explore contaminated environments.

The association between increased medical doctors and ARG and *E. coli* abundance did not hold for older children aged 6–12 months and over 12 months, where the number of medical doctors was negatively associated with both ARG and *E. coli* abundance. These age-dependent patterns may reflect differential antibiotic stewardship practices across income contexts; however, the specific mechanisms remain speculative. In high-resource settings, physicians may adopt more judicious antibiotic prescribing practices for older children with high access to health care^34^. Conversely, it is possible that in low-resource settings, with lower numbers of physicians and reduced access to diagnostics and health care resources, physicians may be more likely to prescribe empiric antibiotic therapy for a sick child in older age categories, increasing ARG carriage^37^. These findings highlight a possible need for targeted behavioural interventions, specifically aimed at physicians, to promote judicious antibiotic use in the fight against AMR.

Income inequality, measured by the Gini index, was a significant socioeconomic variable in our study, showing a significant positive association with both ARG and *E. coli* abundance in children under 3 months of age, as well as with ARG abundance in those aged 3–6 months. A previous study conducted in 15 European countries reported a positive association between the Gini index and rates of AMR for bacteria, including *E. coli*^38^. These data suggest that multi-sectoral action supporting equitable access to health care resources could likely reduce the global burden of AMR.

Antibiotic use is indisputably one of the leading drivers of AMR. We reveal a positive association between defined daily doses of antibiotics and ARG abundance in the <3 months and 3–6 month age groups, as well as with *E. coli* abundance in infants aged 3–6 months and 6–12 months. This aligns with the well-documented evidence that antibiotic exposure disrupts the microbiome, selecting for opportunistic pathogens in children^8,39,40^. Despite the consistency of this association, some have reported an inverse relationship between global antibiotic consumption and resistance, suggesting that broader factors may mediate this relationship^41^. Our finding that national DDD associates the infant resistome, even in a cohort excluding children with recent antibiotic exposure, is intriguing. The finding suggests that national-level DDD indicators are indirectly impacting healthy infants. Possible factors shaping this association could be practices pertaining to IAP, neonatal care, or postnatal horizontal transmission^42^. Higher national DDD might also reflect higher levels of antibiotic exposures in the community and environment, including food and water sources. These antibiotic pressures may elevate ARG carriage in household and community reservoirs, increasing the risk for infant acquisition via contact and facilitating horizontal transmission^43^.

It is important to note that national-level indicators may not capture substantial within-country heterogeneity in these exposures. Studies across multiple continents demonstrate marked subnational variation. In sub-Saharan Africa, C-section rates differ nearly threefold between urban and rural areas^44^. Antibiotic consumption also exhibits considerable regional heterogeneity within countries. In Italy, community antibiotic use in 2018 ranged from 12.7 DDD per 1000 inhabitants per day in northern regions to 20.4 DDD in southern regions^45^. Physician density shows comparable geographic maldistribution. For instance, in most sub-Saharan African countries like Nigeria, rural residents constitute roughly half the population but have access to only about 12% of the country’s physicians, illustrating a substantial concentration of doctors in urban areas despite a predominantly rural population^46^. These within-country differences highlight that national averages may obscure important local variation in exposure contexts that could influence infant resistome development.

Despite its strengths, our study had some limitations. The overrepresentation of studies from HICs constrains the generalisation of our findings to LICs. Therefore, within-age income comparisons should be treated as hypothesis-generating due to the underpowered nature for certain strata such as <3 months in LICs. Additionally, database biases and the lack of comprehensive participant-level metadata on variables such as delivery mode, feeding status, and missing SES variables for some countries hindered deeper exploration of these variables. Critically, our use of national-level indicators (DDD, physician density, Gini index, C-section rates) as contextual variables may limit the generalisability of individual-level inferences from population-level data. These variables represent population exposure contexts rather than direct individual measurements. We also acknowledge limitations on the lack of standardised protocols across the studies regarding sequencing platforms, DNA extraction methods, and sample processing, which could affect the technical comparisons across the datasets. Finally, while ARG carriage reflects potential resistance, it is important to note that carriage does not necessarily equate with infection or phenotypic resistance. While these limitations exist, our findings provide important insights into global ARG dynamics and offer actionable strategies to mitigate AMR risks.

In sum, our findings show that age and socioeconomic factors significantly shape the abundance of ARGs in children, with *E. coli* emerging as a major taxon associated with resistome differences across income groups, particularly in LICs where health care access remains challenging. To mitigate AMR risk, age- and context-specific interventions are most likely to reduce exposure to *E. coli* and ARGs. In young ages and higher-resource settings, efforts that focus on health care strengthening to reduce inequities in access, promote careful use of C-sections, antimicrobial prophylaxis and antibiotic treatment through enhanced health care provider training and development of antimicrobial stewardship programs may help reduce infant carriage of ARGs. In parallel, interventions are needed to protect infants older than 6 months of age in LICs from acquiring ARGs, focusing on drastically reducing fecal exposures through investments in transformative WASH^47^, strengthening of health care systems, specifically expansion of the health care provider workforce, improved infectious disease diagnostics, and antimicrobial stewardship programs. Our findings raise the possibility that, in LMICs where sequencing capacity is limited, routine phenotypic resistance surveillance of *E. coli* could serve as a pragmatic partial indicator of infant ARG burden; however, such surveillance would capture only expressed resistance in this single species and thus should be systematically validated against metagenomic resistome measures in other infant populations. Finally, AMR national action plans must consider the incorporation of age and SES factors into their AMR strategies to create effective and sustainable solutions for populations most at risk.

## Methods

### Search strategy and selection criteria

We aimed to identify studies that used a shotgun metagenomic approach to analyse the gut microbiome in healthy children under the age of two across varying geographic and SES settings. We conducted a systematic search for relevant studies published in EMBASE (1 January 1947 to 29 May 2023), PubMed (1 January 2000 to 29 May 2023), and Web of Science (1 January 1960 to 29 May 2023) using the following search terms: infant, child*, neonat*, metagenomic, gastrointestinal flora, intestinal microbiota, and microbiome. We did not place any restrictions on language. The complete search strategy is in the supplementary file (Supplementary Table 1). Additionally, we conducted an advanced search in public data repositories to complement our systematic search. Searches were conducted in NCBI-BioProject and European Molecular Biology Laboratory’s European Bioinformatics Institute (EMBL-EBI), on 29 May 2023, for infant metagenomic BioProjects using the following terms: infant, microbiome, metagenomic, and resistome. We defined a BioProject as a collection of biological data related to a single research initiative, typically from one organisation or consortium. BioSamples provide descriptive information about the biological source material used in the experiment.

Studies were included if they reported metagenomic sequencing of faecal samples or rectal swabs from healthy full-term human infants aged 0–2 years, with no record of antibiotic exposure prior to study inclusion, and made their metagenomic data available. BioProjects were excluded if they did not have samples in the 0–2 years range, only had 16S rRNA sequenced reads, were derived from populations with extreme or unique lifestyle contexts, such as hunter-gatherer communities, only had samples from antibiotic-exposed or sick infants, lacked age-specific or accompanying metadata in general. The identified articles were screened using Rayyan, a semi-automated web-based screening tool^48^. Two assessors conducted the screening independently, and a third assessor was engaged to resolve any differences in the selected studies.

### Variable identification

Using variables from the World Bank and World Health Organisation (WHO), we next identified the following variables for each fecal sample using nationally-aggregated data: Domestic general government health expenditure per capita (in US dollars), percent of the population practising open defecation, population using safely managed sanitation services, percent of people using at least basic drinking water services, percent of population using at least basic sanitation services, under-five mortality rate, defined daily dose of antibiotics (DDD) (per 1000 population), number of medical doctors (per 10,000 population), and Gini index (social inequality index). Data on country caesarean section (C-section) as a percent of live births were obtained from World Population Review^49^. The values for these variables were obtained for the year of sample collection, which was determined by screening the methods section of the included articles. Where absent, the sequencing read archive (SRA) collection date was used as the year of sample collection. If both were unavailable, an estimate was made based on the year closest to the publication date. Specifically for samples collected in Ethiopia^50^ and Mozambique^51^, where the year of sample collection was unavailable, an estimate was made by averaging data less than or greater than 3 years from the year of publication. If no data on the year of sample collection were available beyond the 6-year range, that specific country-level variable was recorded as missing.

Based on data from the World Bank, countries were classified into their respective income groups according to the year of sample collection: low-income country (LIC), LMIC, UMIC, and high-income country (HIC) (Supplementary Table 2).

### Metagenomic read processing

Raw metagenomic reads were downloaded from EMBL-EBI and SRA with a script that downloaded FASTQ files using their file transfer protocol uniform resource locators. The downloaded reads were processed using BBDuk (v29.01) and BBMap (v29.01)^52^. The raw reads were trimmed to remove adapters, while bases with a quality score lower than 15 and reads shorter than 45 bps were discarded. Reads mapping to the reference human genome were removed, and the remaining reads were merged into longer, single reads using the read-pairing tool BBMerge (v29.01) (min read overlap = 16). FastQC (v0.12.1) was used to check the quality of the merged reads (https://www.bioinformatics.babraham.ac.uk/projects//fastqc/).

The Resistome Gene Identifier (RGI) tool (v6.0.3) was used to map the quality-controlled reads against the Comprehensive Antibiotic Resistance Database (v3.2.8) for resistome profiling^53^. ARG detection included both ‘Perfect’ and ‘Strict’ hits, while ‘Loose’ hits were excluded to ensure high-confidence ARG identification. To account for differences in the sequencing depth and read length, the obtained resistance gene counts were normalised to reads per kilobase per million mapped reads (RPKM) values. Specifically, for each sample, the total number of mapped reads was obtained from the overall mapping statistics file, which is an output from RGI. RPKM was calculated using the formula:

$${RPKM}=\frac{{ARG}-{mapped}\,{reads}\,X\,{10}^{9}\,}{{Reference}\,{sequence}\,{length}\,\left({bp}\right)\,X\,{Total}\,{mapped}\,{reads}}$$

where ARG-mapped reads represent the number of reads mapping to each detected ARG, reference sequence length was extracted from the CARD database entry for each ARG as reported in the RGI gene mapping output, and total mapped reads represent the total number of quality-controlled reads per sample that mapped to CARD reference sequences^54^.

Because analyses were performed on unassembled short-read metagenomic data, we did not attempt to distinguish plasmid-encoded from chromosomally encoded ARGs. Genomic querying and taxonomic profiling were conducted using the Sylph tool (v0.6.1)^55^. Specifically, the ‘sylph sketch’ function was used to break the reads into k-mers. The sketched reads were then analysed using the ‘sylph profile’ flag against the Genome Taxonomy Database (GTDB-R214). The obtained abundances were used in the subsequent taxonomic analysis.

### Faecal taxonomic composition across age and income

All analyses were conducted in R version 4.3.1. We performed species and genus-level analyses to explore the distribution of taxa across income categories by age subgroups (<3, 3–6, 6–12, and >12 months). Zeros were replaced using the Bayesian multiplicative method in the zCompositions version 1.5.0-5^56^, after which the abundance data were transformed to centred log-ratio (CLR) using the compositions package version 2.0-8. We chose CLR because it allows all samples and taxa to be compared in the same log-ratio space, is compositionally aware, and is easier to interpret^57^. Subsequent statistical analyses were performed on the pairwise income group comparisons using Dunn’s test. We subsequently controlled the family-wise error rate across the set of pairwise Dunn tests using Holm’s procedure, which does not assume independence among tests and is uniformly more powerful than single-step Bonferroni, providing a conservative yet efficient adjustment for our limited set of planned comparisons^58^. Correlations between resistome abundance and species were carried out using the Spearman coefficient in microViz version 0.12.1^59^.

### Resistome composition and gene abundance across geographies

ARG abundance was compared across income categories and age subgroups using the Kruskal–Wallis test. Wilcoxon’s test assessed pairwise differences, with significant *p*-values (*p* < 0.05) adjusted using the Holm method. We assessed alpha diversity of the resistome (Shannon, Inverse Simpson, and ARG richness) across age and income. Resistome beta-diversity was computed across income groups, feeding method, delivery mode, and age using principal coordinate analysis utilising the Bray–Curtis dissimilarity index. A Permutational Multivariate Analysis of Variance (PERMANOVA) with 9999 permutations was carried out using vegan version 2.6-4 with correction for country in the formula to test for any significant differences between groups in the ordinations. We also carried out unsupervised machine learning, *k*-means clustering, to investigate any clustering in the distance matrix. We chose *k*-means as a straightforward, non-parametric way to partition the samples into clusters^60^. *K*-means has the advantage of being computationally efficient even with our large sample size (*n* = 1944). To determine the optimal number of clusters, we evaluated *k* = 1 to *k* = 10 using gap statistic and silhouette analysis^61,62^. The gap statistic compares within-cluster dispersion to that expected under a null reference distribution, with higher values indicating stronger clustering structure. Silhouette analysis quantifies how well samples fit within their assigned clusters, with values ranging from −1 to +1.

We performed a sensitivity analysis to evaluate the robustness of our clustering findings by comparing *k*-means clustering results from Bray–Curtis dissimilarity (primary analysis) with clustering derived from Jaccard distance on the same samples. We also assessed cluster robustness by evaluating stability across 100 iterations with different random seed initialisations and calculated the adjusted Rand index (ARI) to quantify assignment consistency. Infants lacking data on feeding practices or delivery mode were omitted from the corresponding subgroup comparisons.

### Resistome and microbiome associations

To quantify concordance between microbiome taxonomic composition and resistome structure, we carried out Procrustes analysis, using the vegan package (version 2.6-4) with 999 permutations. Bray–Curtis dissimilarity matrices were calculated separately for taxonomic abundances and ARG abundances, followed by principal coordinate analysis on each matrix. Procrustes rotation was applied to optimally superimpose the two ordinations, and concordance was assessed using the Procrustes correlation coefficient (*r*) and sum of squared residuals (*M*²). Analyses were performed on the entire dataset and stratified by income group to evaluate setting-specific patterns.

### Associations between ARG and *Escherichia coli* abundance with national-level variables

To test the association between ARG and *E. coli* abundance and national-level factors in our dataset, we used a cross-fit partialling-out model with tenfolds to estimate the effect of the variables on both ARG and *E. coli* abundance. Cross-fit partialling-out estimation methods overcome overfitting by orthogonalising the relevant estimators^63^. To reduce multicollinearity and improve model interpretability, we excluded highly correlated variables (|*r*| > 0.8), as shown in the clustered correlation matrix (Supplementary Fig. 1). The final model retained four key predictors: defined daily dose of antibiotics (DDD), number of medical doctors, Gini index (social inequality index), and C-section rates, all of which had acceptable multicollinearity (VIF < 5). Country and income grouping were included as fixed-effect controls in the final model. To ensure no single variable dominated the regression, we standardised all predictors to have a mean of zero and a standard deviation of one. Given the high dimensionality of our dataset, we opted to use Lasso for penalisation to improve model stability and reduce overfitting. Lasso was used solely for regularisation, not for variable selection. Finally, we employed robust standard errors, clustered by country. This analysis was run on the entire dataset, including age as a variable, and repeated separately within each defined age subgroup to assess potential age-related differences.

## Supplementary information

Supplementary information

## Data Availability

The study used publicly available datasets with corresponding BioProject numbers in the main text.
