# Supplementary material for: Distinct infant resistome trajectories shaped by country income and geography revealed through global metagenomics reanalysis
Source: NPJ Antimicrob Resist. 2026 Apr 15;4:24. doi: 10.1038/s44259-026-00194-8 (PMC13083862; doi:10.1038/s44259-026-00194-8)
Supplement: Supplementary file 1 — Supplementary information [file 44259_2026_194_MOESM1_ESM.docx]

**Supplementary Information**

**Income and geography shape antimicrobial resistance gene carriage in children under two: An individual participant pooled data re-analysis of gut metagenomes across global settings**

Charlie C Luchen^1,2^, Gonçalo J Piedade^1^, Mwelwa Chibuye^1,2^, Michelo Simuyandi^2^, Caroline C Chisenga^2^, Roma Chilengi^4,5^, Samuel Bosomprah^2,3^, Constance Schultsz^1,6,7^, Daniel R Mende^6,7,†^, Vanessa C Harris^1,7,8,†^

^†^Shared senior authorship

Affiliations:

^1^ Amsterdam UMC, location University of Amsterdam, Department of Global Health, Amsterdam Institute for Global Health and Development, Meibergdreef 9, Amsterdam, the Netherlands

^2^ Research Division, Centre for Infectious Disease Research in Zambia, Lusaka, Zambia

^3^ Department of Biostatistics, School of Public Health, University of Ghana, Accra.

^4^ Zambia National Public Health Institute, Ministry of Health, Lusaka 10101, Zambia

^5^ Republic of Zambia State House, Lusaka 10101, Zambia

^6^Amsterdam UMC, location University of Amsterdam, Department of Medical Microbiology, Meibergdreef 9, Amsterdam, the Netherlands

^7^ Amsterdam Institute of Infection and Immunity, Infectious Diseases, Amsterdam University Medical Center, Amsterdam, the Netherlands

^8^ Amsterdam UMC, location University of Amsterdam, Department of Internal Medicine, Division of Infectious Diseases, Meibergdreef 9, Amsterdam, the Netherlands

# **Supplementary Table 1**: search strategy

| **Embase Classic+Embase <1947 to 2023 May 29>** | |  |  |  |  |
| --- | --- | --- | --- | --- | --- |
| **#** | **Query** | **Results** |  |  |  |
| 1 | exp infant/ | 1,313,269 |  |  |  |
| 2 | exp child/ | 3,503,286 |  |  |  |
| 3 | neonate.mp. or exp newborn/ | 707,645 |  |  |  |
| 4 | exp antibiotic resistance/ or antimicrobial resistance gene.mp. | 221,656 |  |  |  |
| 5 | exp drug resistance/ or antibiotic resistance gene*.mp. | 390,637 |  |  |  |
| 6 | exp microbiome/ or microbiome.mp. or exp bacterial microbiome/ | 76,770 |  |  |  |
| 7 | gut microbiota.mp. or exp intestine flora/ | 106,837 |  |  |  |
| 8 | gastrointestinal microbio*.mp. | 2,398 |  |  |  |
| 9 | exp metagenomics/ | 28,022 |  |  |  |
| 10 | 1 or 2 or 3 | 3,513,815 |  |  |  |
| 11 | 4 or 5 or 6 or 7 or 8 | 538,794 |  |  |  |
| 12 | 9 and 10 and 11 | 1,035 |  |  |  |
| **Web of science** |  |  |  |  |  |
| **#** | **Search Query** | **Database** | **Results** | **Date Run** |  |
| 1 | ((((ALL=(infant)) OR ALL=(neonate))) OR ALL=(newborn)) OR ALL=(child*) | Web of Science Core Collection | 3627734 | Mon May 29 2023 21:29:51 GMT+0200 (Central European Summer Time) |  |
| 2 | ((((ALL=(antimicrobial resistance)) OR ALL=(antibiotic resistance)) OR ALL=(antibiotic resistance gene)) OR ALL=(drug resistance)) OR ALL=(resistance gene) | Web of Science Core Collection | 660131 | Mon May 29 2023 21:32:30 GMT+0200 (Central European Summer Time) |  |
| 3 | (((ALL=(microbiome)) OR ALL=(intestinal flora)) OR ALL=(gastrointestinal microbiota)) OR ALL=(gut microbiome) | Web of Science Core Collection | 98267 | Mon May 29 2023 21:34:10 GMT+0200 (Central European Summer Time) |  |
| 4 | ALL=(metagenomics) | Web of Science Core Collection | 14390 | Mon May 29 2023 21:34:44 GMT+0200 (Central European Summer Time) |  |
| 5 | #2 OR #3 | Web of Science Core Collection | 750706 | Mon May 29 2023 21:35:01 GMT+0200 (Central European Summer Time) |  |
| 6 | #1 AND #4 AND #5 | Web of Science Core Collection | 602 | Mon May 29 2023 21:35:14 GMT+0200 (Central European Summer Time) |  |
| **PubMed** |  |  |  |  |  |
| **Search number** | **Query** | **Sort By** | **Filters** | **Results** | **Time** |
| 1 | (((((infant) OR (child*)) OR (neonate)) OR (newborn)) AND (metagenomics)) AND ((((((antibiotic resistance) OR (antimicrobial resistance)) OR (antimicrobial resistance gene)) OR (antibiotic resistance gene)) OR (drug resistance)) OR (((((microbiome) OR (gut microbiota)) OR (gut microbiome)) OR (intestinal floral)) OR (gastrointestinal microbiome))) |  | Humans, Newborn: birth-1 month, Infant: birth-23 months, Infant: 1-23 months, Exclude preprints | 638 | 14:54:13 |

# = search number


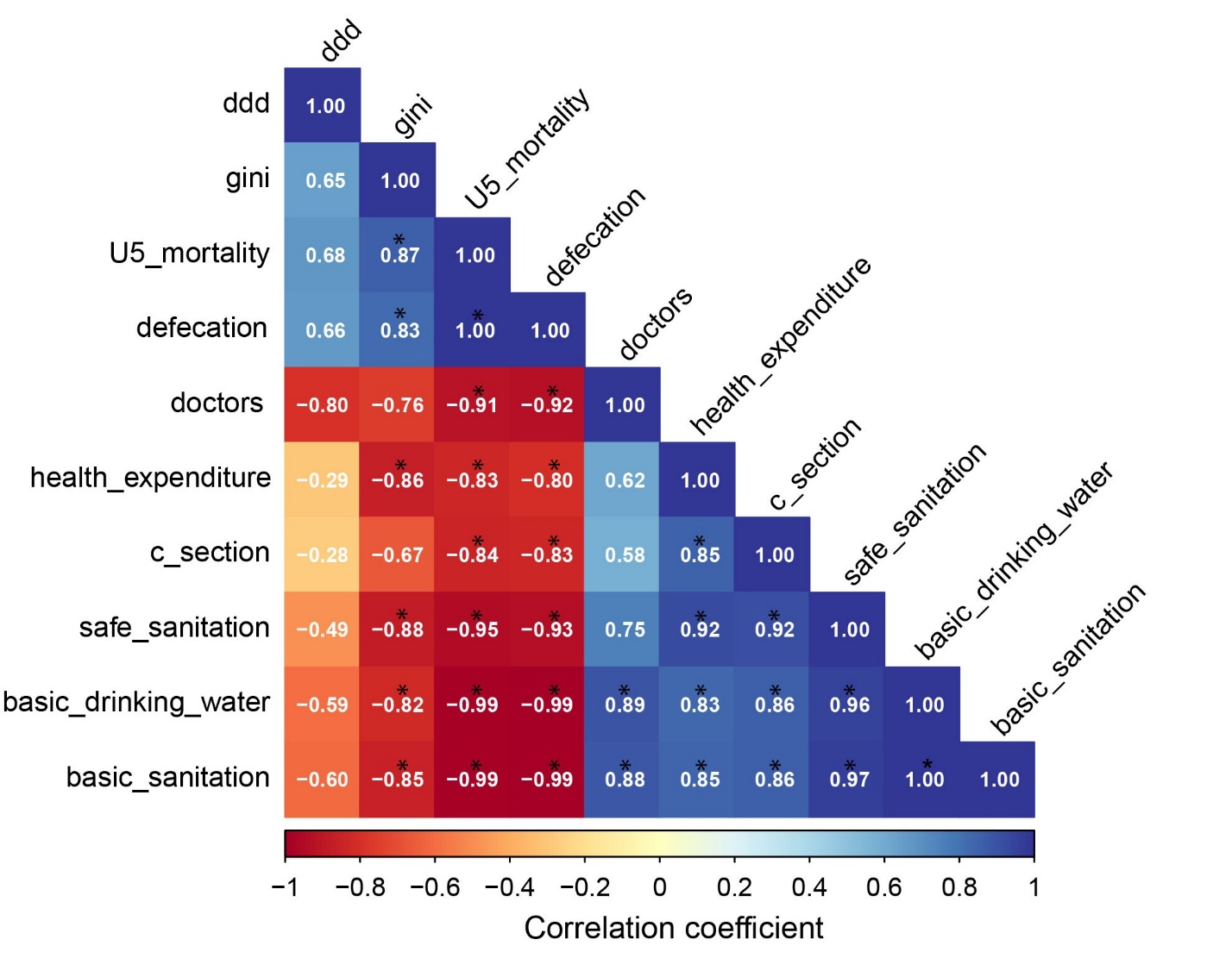


**Supplementary Fig. 1: Correlation heatmap of the identified nationally aggregated variables, clustered by similarity**. The dark blue indicates strong positive correlations, while dark red indicates strong negative correlations. Only the upper triangle of the matrix is shown to improve readability. Strongly collinear variable pairs (defined as r > 0.8) are marked with an asterisk (*). Variables include defined daily dose (ddd), under-5 mortality (U5_mortality), gini index (gini), percent of the population practising open defecation (defecation), number of medical doctors per 10, 000 population (doctors), domestic general government health expenditure per capita (health_expenditure), cesarean section births (c_section), population using safely managed sanitation services (safe_sanitation), percent of people using at least basic drinking water services (basic_drinking_water), percent of population using at least basic sanitation services (basic_sanitation).

**Supplementary Table 2**: Infant feeding method, delivery mode, and national-level health and socioeconomic indicators by country and income group

|  | | **Feeding Method, n (%)** | | | | **Delivery Mode, n (%)** | | | **National-Level Variables** | | | | |
| --- | --- | --- | --- | --- | --- | --- | --- | --- | --- | --- | --- | --- | --- |
| **Income/Country** | **N** | **Breastfed** | **Formula** | **Mixed** | **Missing(a)** | **Vaginal** | **C-section** | **Missing (b)** | **DDD(c)** | **Physicians (d)** | **Gini (e)** | **C-section (f)** |  |
| **Low** | | | | | | | | | | | | | |
| Ethiopia (Tett et al., 2019) | 14 | NA | NA | NA | 14 (100.0) | 14 (100.0) | - | - | 10.51 | 0.76 | 35 | 1.9 |  |
| Mozambique (Kim et al., 2022) | 91 | NA | NA | NA | 91 (100.0) | NA | NA | 91 (100.0) | NA | 0.76 | 52.25 | 3.9 |  |
| **Lower Middle** | | | | | | | | | | | | | |
| Zimbabwe (Robertson R.C. et al., 2022) | 319 | 188 (58.9) | 2 (0.6) | 4 (1.3) | 125 (39.2) | 292 (91.5) | 20 (6.3) | 7 (2.2) | 25.5 | 1.36 | 43.65 | 5.8 |  |
| **Upper Middle** | | | | | | | | | | | | | |
| Russia (Vatanen T. et al., 2016) | 104 | 4 (3.8) | - | 3 (2.9) | 97 (93.3) | NA | NA | 104 (100.0) | 14.82 | 44.5 | 40.3 | 13 |  |
| **High** | | | | | | | | | | | | | |
| Estonia (Vatanen T. et al., 2016) | 76 | 14 (18.4) | 1 (1.3) | 3 (3.9) | 58 (76.3) | NA | NA | 76 (100.0) | 11.6 | 32 | 31.8 | 20.3 |  |
| Finland (Vatanen T. et al., 2016) | 67 | 17 (25.4) | - | 11 (16.4) | 39 (58.2) | NA | NA | 67 (100.0) | 19.6 | 36 | 27.7 | 16.4 |  |
| Germany (Korpela K. et al., 2018) | 11 | NA | NA | NA | 11 (100.0) | 11 (100.0) | - | - | 13.33 | 41 | 30.9 | 30.5 |  |
| Italy (Asnicar et al., 2017) | 8 | 6 (75.0) | - | 2 (25.0) | - | NA | NA | 8 (100.0) | 24.5 | 39 | 35.4 | 35 |  |
| Luxembourg (Korpela K. et al., 2018) | 8 | NA | NA | NA | 8 (100.0) | 8 (100.0) | - | - | 23.2 | 29 | 31.2 | 30.5 |  |
| Sweden (Bäckhed et al., 2015) | 202 | 100 (49.5) | 6 (3.0) | 20 (9.9) | 76 (37.6) | 175 (86.6) | 27 (13.4) | - | 15.3 | 37.5 | 27.7 | 17.4 |  |
| UK (Shao et al., 2019) | 918 | 359 (39.1) | 158 (17.2) | 319 (34.7) | 82 (8.9) | 461 (50.2) | 457 (49.8) | - | 20.03 | 27.78 | 33 | 27.8 |  |
| USA (Casaburi G. et al., 2021) | 126 | 57 (45.2) | 28 (22.2) | 40 (31.7) | 1 (0.8) | NA | NA | 126 (100.0) | NA | 25.7 | 41.4 | 32 |  |

(a) Missing = BioSamples with missing feeding method data. NA indicates all samples lack feeding data; (b) Missing = BioSamples with missing delivery mode data. NA indicates all samples lack delivery data; (c) DDD = Defined daily dose of antibiotics per 1,000 population obtained from the World Health Organisation, NA indicates missing data; (d) Physicians = Number of medical doctors per 10,000 population obtained from the World Health Organisation; (e) Gini = Gini index (income inequality measure, 0-100) obtained from the World Bank; (f) C-Section Rate = Percentage of live births, obtained from world population review.


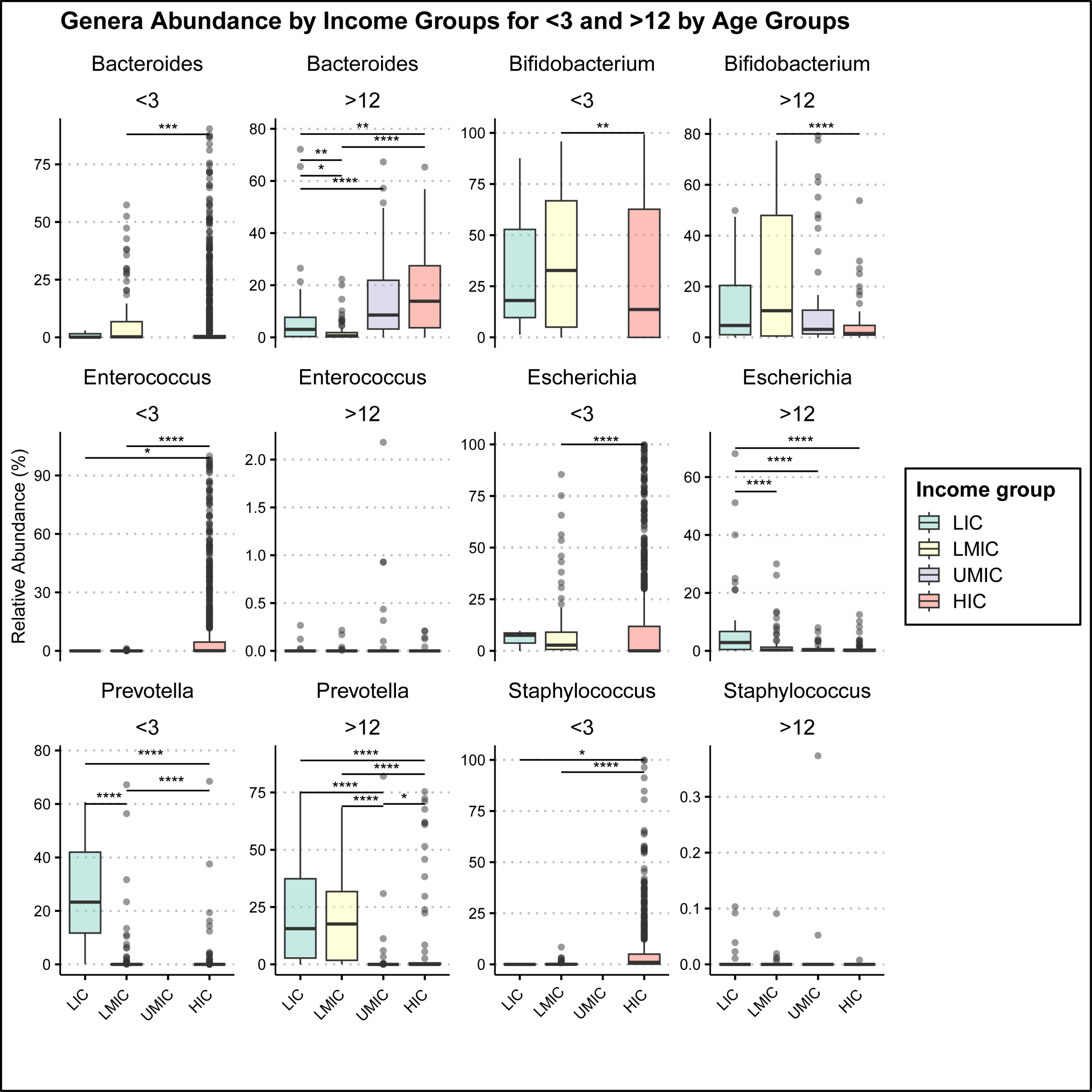


**Supplementary Fig. 2: Relative Abundance of Genera by Income Groups and Age Categories (<3 and >12 months).** Boxplots display the relative abundance (%) of bacterial genera across different income groups (LIC, LMIC, UMIC, and HIC) for two age categories (<3 months and >12 months). Sample sizes by income group: <3 months: LIC n=3, LMIC n=105, HIC n=1104; >12 months: LIC n=47, LMIC n=104, UMIC n=57, HIC n=76. Due to the large number of species, only a few genera that were of interest in Figure 2B are shown. Statistical comparisons between income groups were performed using Dunn's test for multiple pairwise comparisons, with p-values adjusted using the Holm method to control for family-wise error rates. Only significant differences are shown. * *p* < 0.05, ** *p* < 0.01, *** *p* < 0.001, **** *p* < 0.0001.


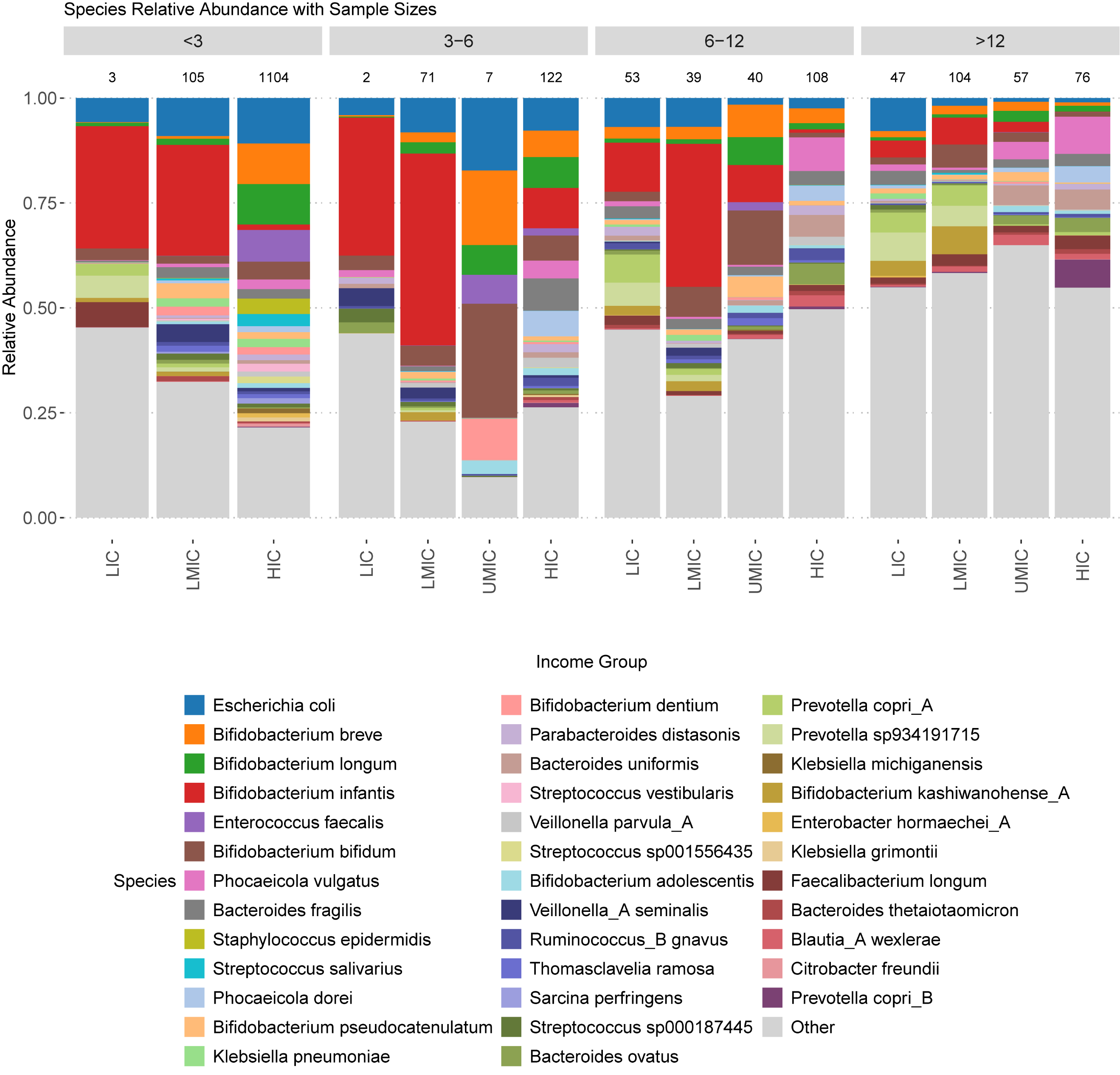


**Supplementary Fig. 3: Relative Abundance of Species by Income Groups and Age Categories.** Only the 37 most abundant species are shown, while the rest are grouped as ‘other’. Number annotations on top of the bars represent the sample sizes in that income group.


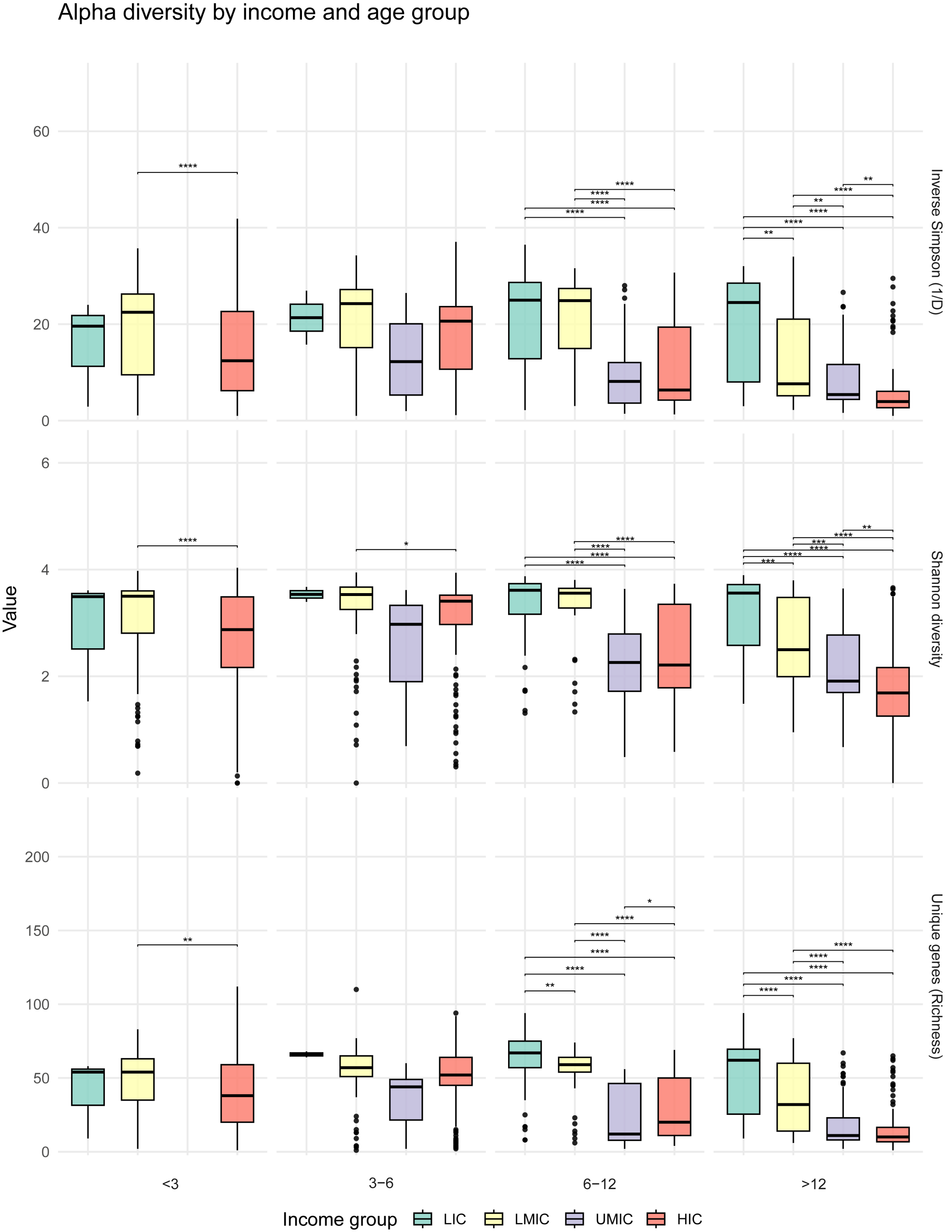


**Supplementary Fig. 4: Alpha diversity indices by income groups and age groups.**

Only significant associations (*p* < 0.05) following Holm correction are shown, * *p* < 0.05, ** *p* < 0.01, *** *p* < 0.001, **** *p* < 0.0001. Sample sizes by income group: <3 months: LIC n=3, LMIC n=105, HIC n=1104; 3-6 months: LIC n =2, LMIC n=71, UMIC n=7, HIC n=122; 6-12 months: LIC n=53, LMIC n=39, UMIC n=40, HIC n=108; >12 months: LIC n=47, LMIC n=104, UMIC n=57, HIC n=76.


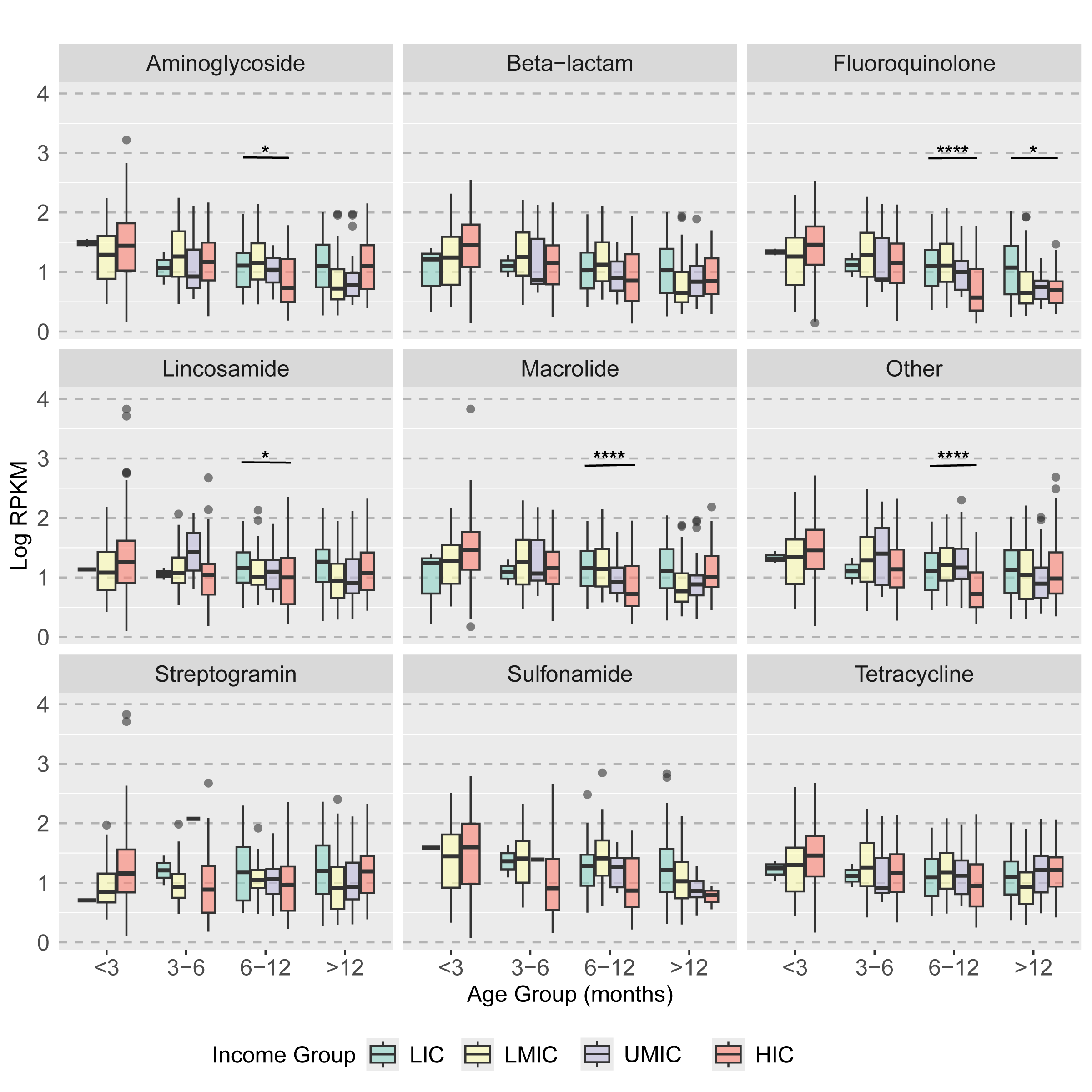


**Supplementary Fig. 5: Drug class abundance distribution by income group over time.**

The boxplots show the log 10-transformed relative abundance (RPKM) of antimicrobial resistance gene (ARG) classes stratified by age groups (< 3 months, 3–6 months, 6–12 months, and > 12 months) and income groups (Low, Lower Middle, Upper Middle, and High). Given the large number of comparisons, p values were only computed between Low and High income and only the significant associations (*p* < 0.05) following Holm correction are shown, * *p* < 0.05, **** *p* < 0.0001.


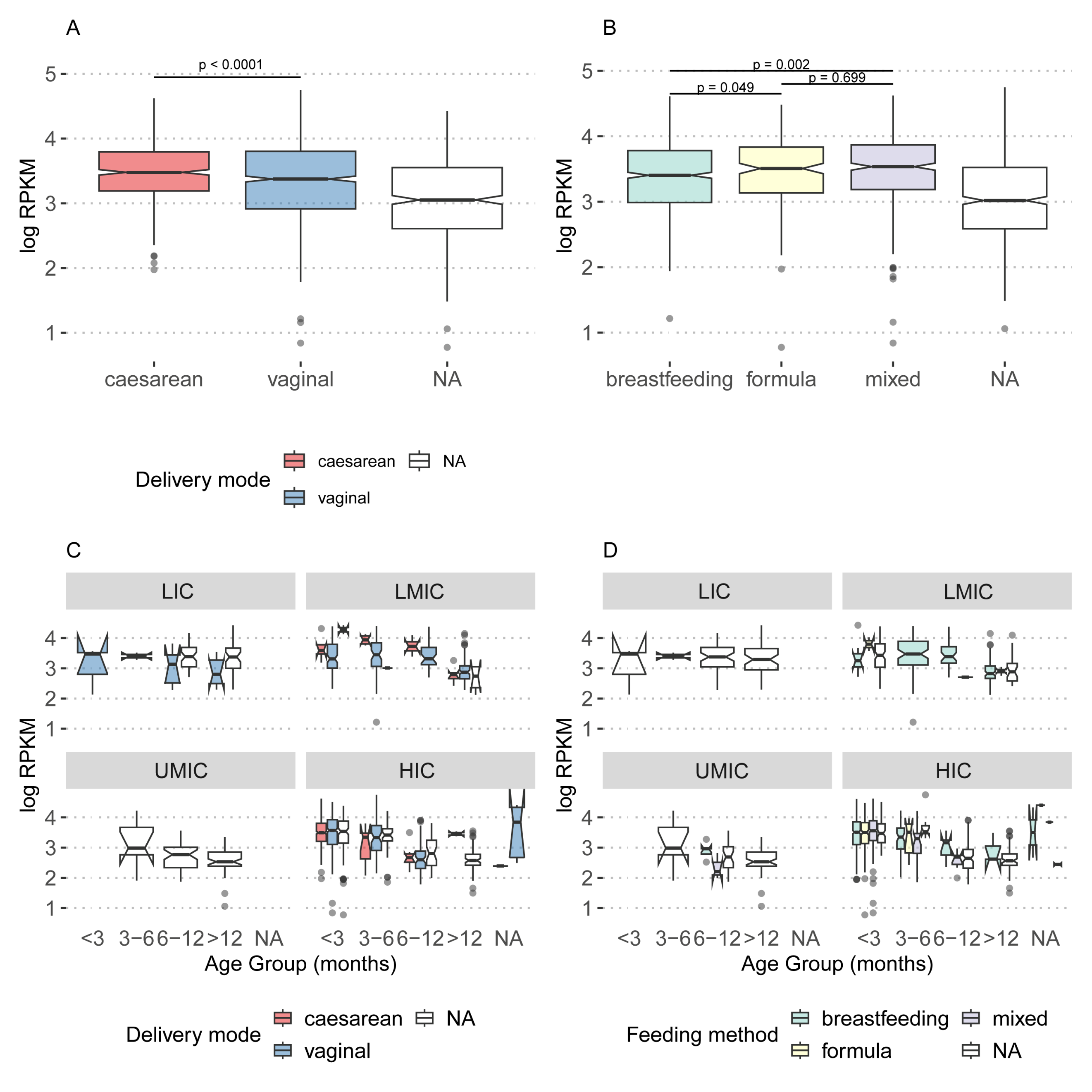


**Supplementary Fig. 6: ARG abundance by feeding method and delivery mode. A** Shows ARG abundance comparisons by delivery mode across the entire dataset, (**B**) stratifies this relationship by age and income grouping. **C** Indicates ARG abundance by feeding method across the dataset while (**D**) stratifies this relationship by age and income group. NA indicates missing data. Associations with Holm adjustment following Wilcoxon’s test are shown in (**A**) and (**C**). Samples with missing metadata are displayed as "NA" for transparency but were not included in statistical comparisons. Additional information on the sample sizes in the group is found in the Supplementary Table 2.


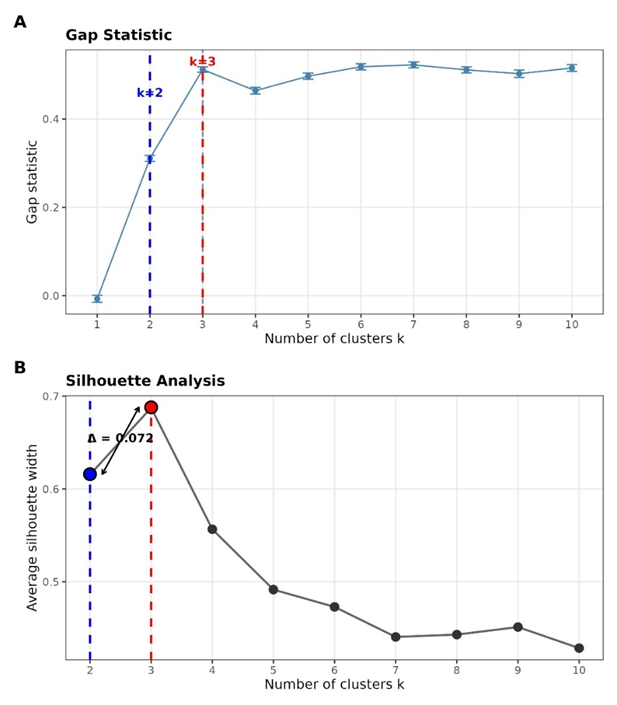


**Supplementary Fig 7**: Determination of optimal cluster number for resistome K-means clustering. **A** Gap statistic value plotted against the number of clusters (k = 1 to 10). (**B**) Average silhouette width values plotted against the number of clusters (k = 2 to 10), the highest silhouette width is observed at k=3 (red circle, average silhouette width = 0.688), followed closely by k=2 (blue circle, average silhouette width = 0.616), Δ indicates the difference between k=2 and k=3 (Δ = 0.072).


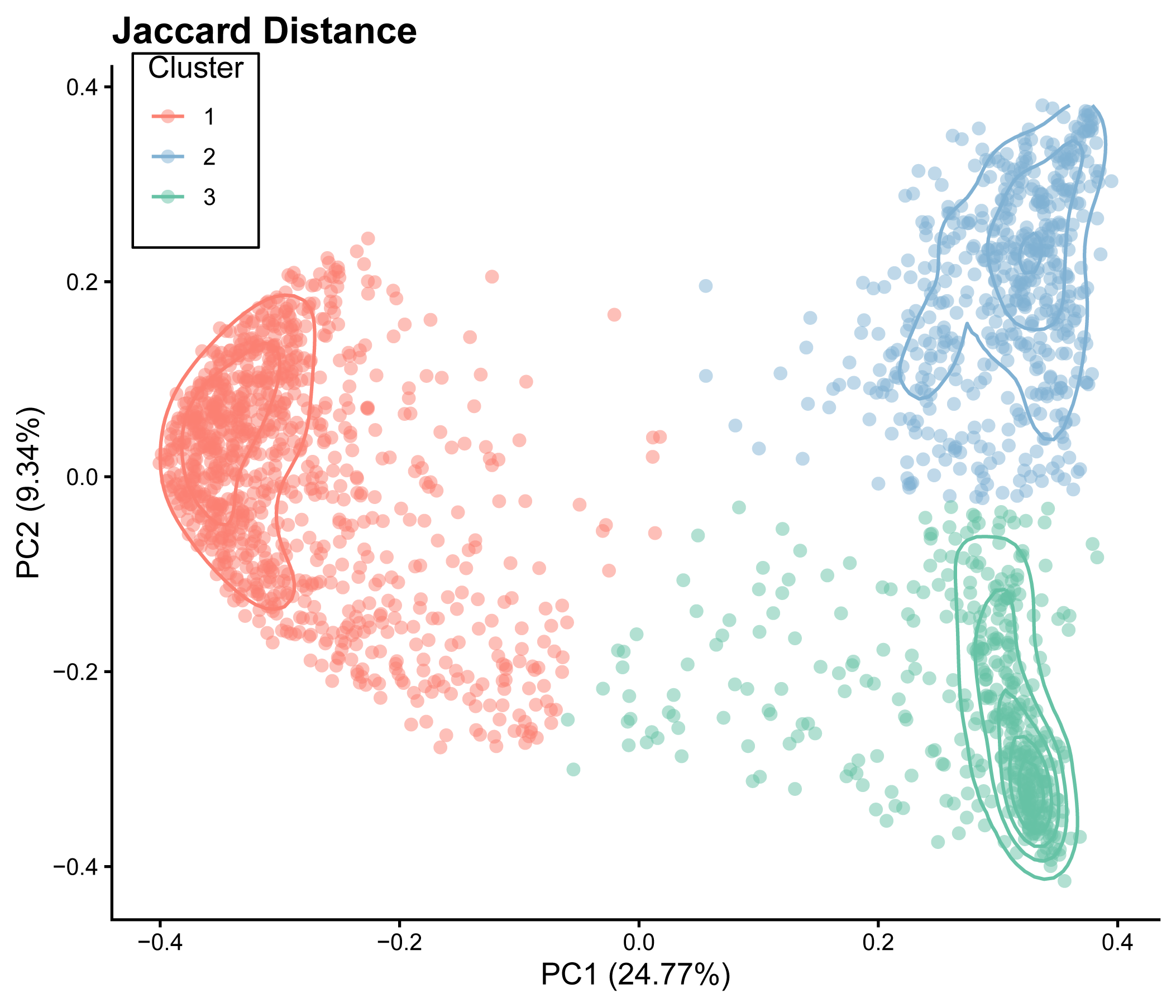


**Supplementary Fig 8**. Principal coordinate analysis of resistome beta-diversity with k-means clustering overlay using Jaccard distances. Density contours indicate sample concentration within each cluster.

**Supplementary Table 3:** K=3 cluster agreement between Bray-Curtis and Jaccard distance

| **Metric** | **Value** |
| --- | --- |
| Total samples analysed | 1944 |
| Overall agreement (%) | 99.1 |
| Adjusted Rand Index (ARI) | 0.974 |
| Cluster 1 concordance (%) | 98.4 |
| Cluster 2 concordance (%) | 100.0 |
| Cluster 3 concordance (%) | 99.8 |


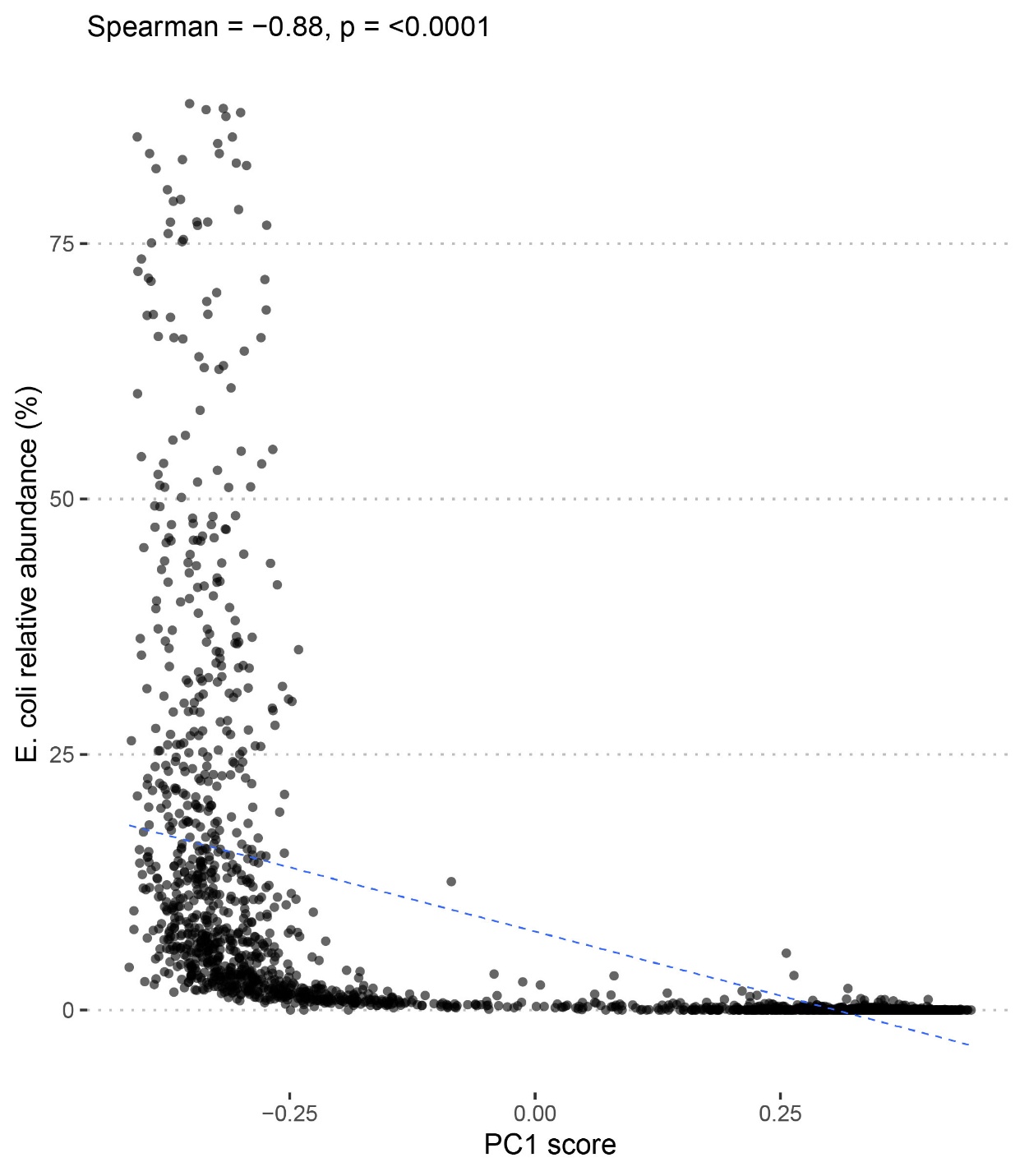


**Supplementary Fig. 9: Correlation between Principal component 1 (PC1) scores and *E. coli* abundance**. Each black point represents one sample


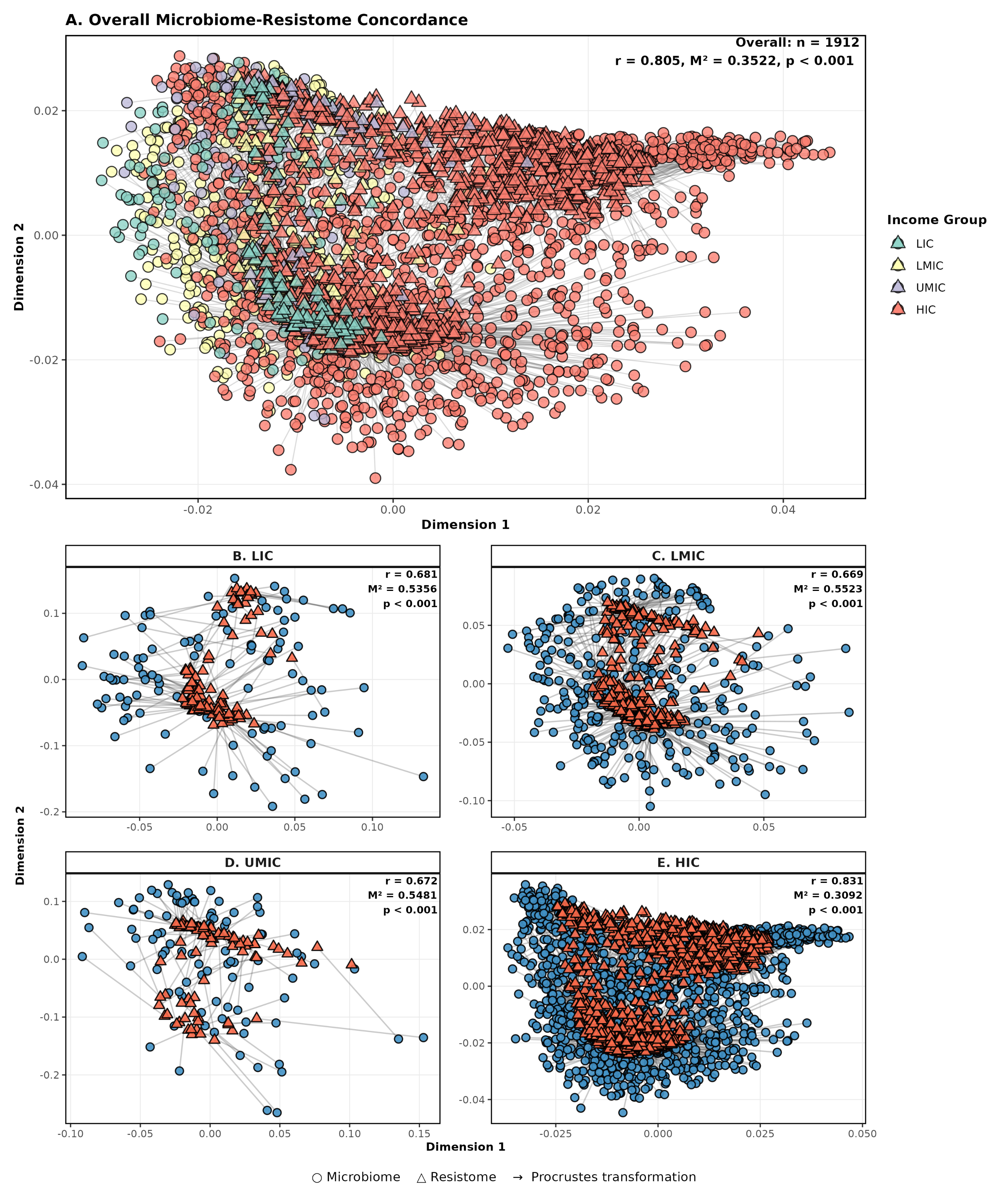


**Supplementary Fig 10: Procrustes analysis showing concordance between microbiome composition and resistome profiles. (A)** Overall Procrustes superimposition of microbiome and resistome ordinations for all samples, colored by country income classification (LIC = low-income countries; LMIC = lower-middle-income countries; UMIC = upper-middle-income countries; HIC = high-income countries). Circles represent microbiome ordination coordinates, triangles represent resistome ordination coordinates, and gray arrows indicate the Procrustes transformation for each sample pair. The correlation coefficient (r), sum of squared residuals (M²), and statistical significance are shown. (**B-E**) Income group-stratified Procrustes analyses showing microbiome-resistome concordance within LIC (B), LMIC (C), UMIC (D), and HIC (E) settings. Blue circles denote microbiome ordination positions, orange-red triangles denote resistome ordination positions, and gray arrows connect paired coordinates for each sample.

**
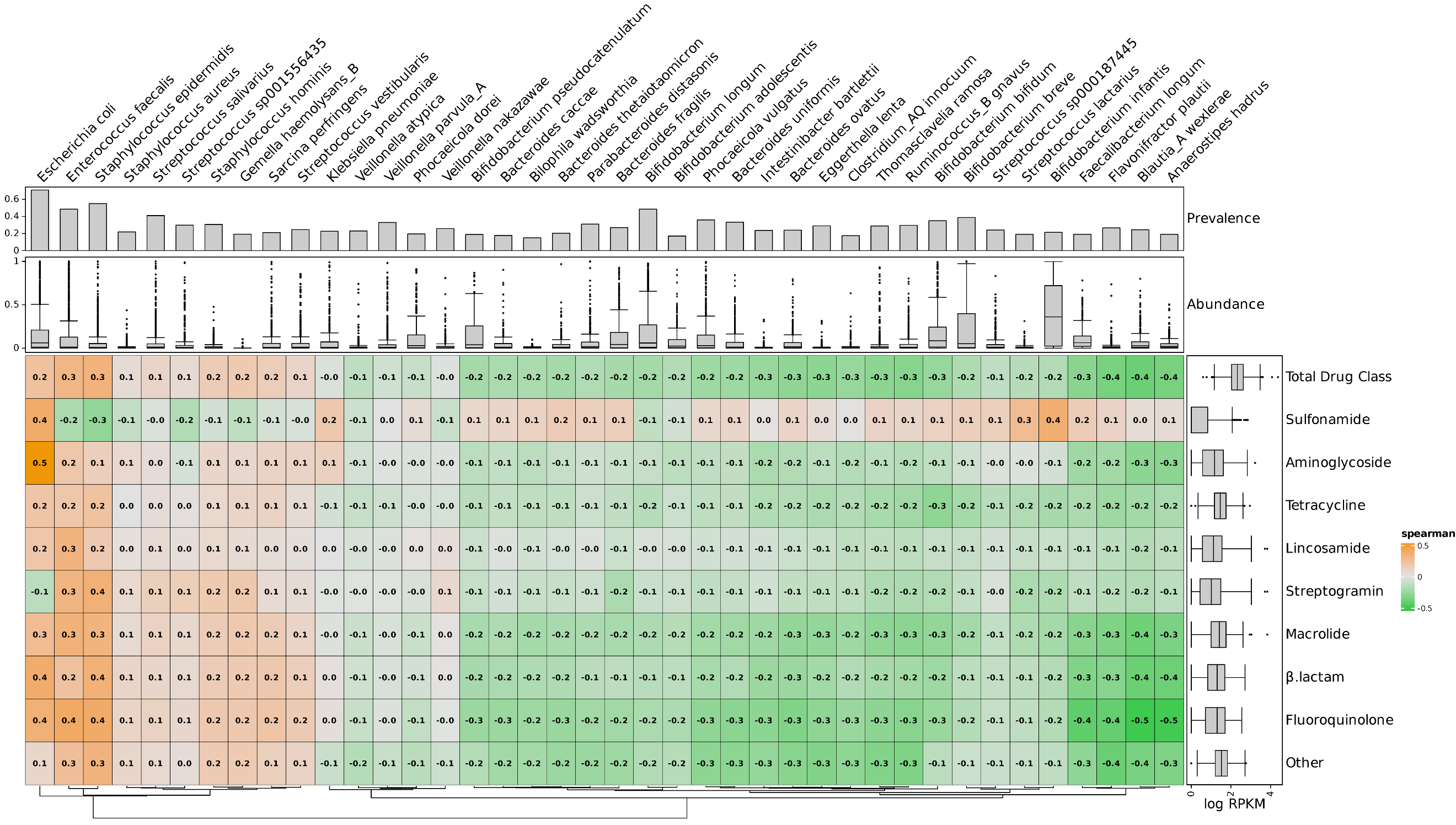
**

**Supplementary Fig. 11: Correlation heatmap testing the relationship between taxonomic and ARG abundance.** The heatmap displays the Spearman correlation coefficients (ranging from -0.5 to 0.5) between the relative abundances of bacterial genera (columns) and ARG classes (rows). Positive correlations are represented in green, while negative correlations are represented in orange, with the intensity of the colour and the text annotation indicating the strength of the correlation.


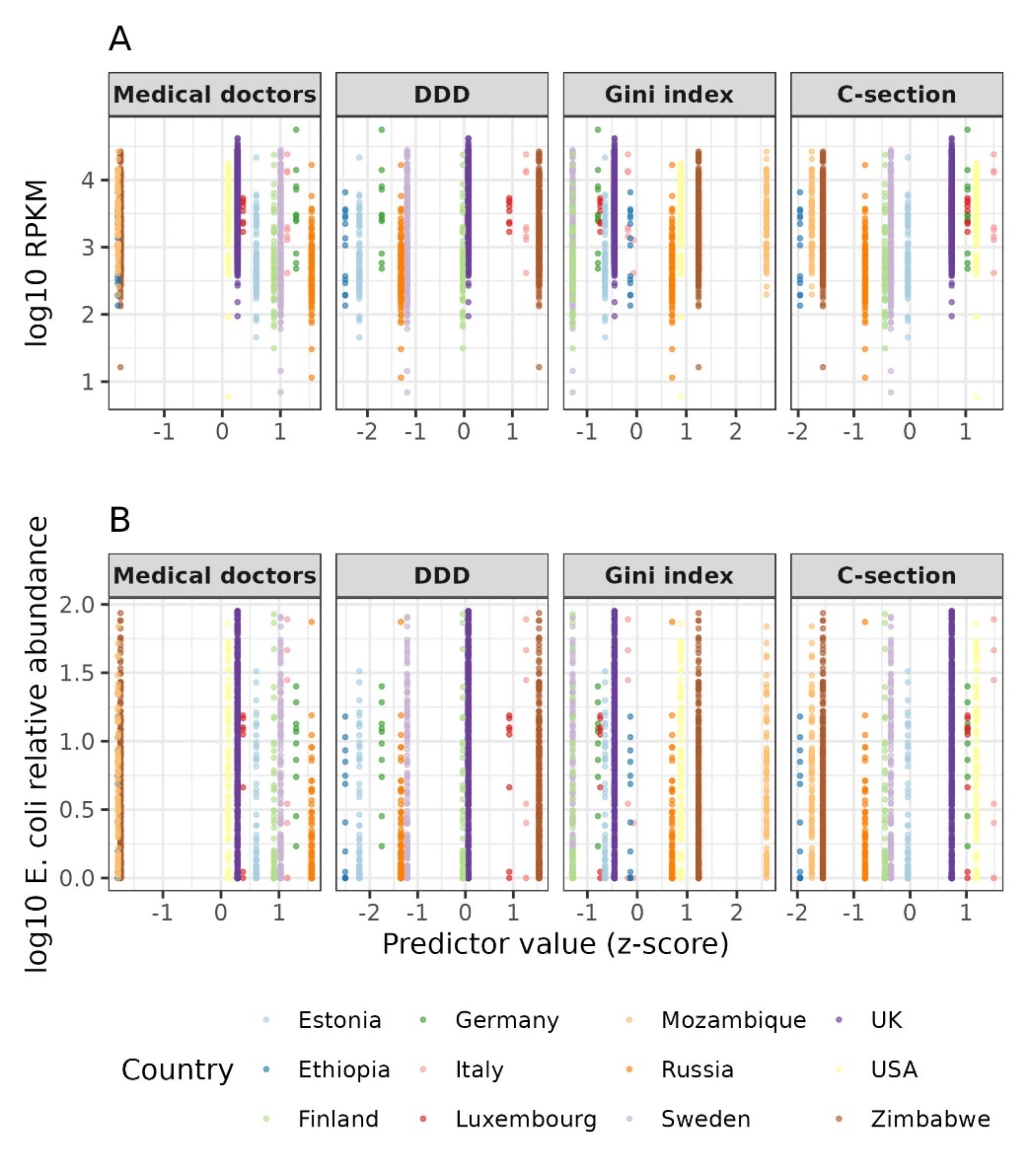


**Supplementary Fig. 12: (A)** Scatterplot showing the distribution of ARGs across the four different z-scored country-level variables that made it to the final model. **(B)** The same predictors plotted against *E. coli* relative abundance. Points represent BioSamples and colours show the different countries. Predictors were standardised (mean = 0, standard deviation. = 1).

# **Supplementary Table 4**: Association of ARG and *E. coli* abundance with age and national-level variables

| ARG abundance | |  | *E. coli* abundance | |
| --- | --- | --- | --- | --- |
| Variable | **Difference in mean log ARG abundance (95% CI)** | ***P* -value** | **Difference in mean log *E. coli* abundance (95% CI)** | ***P* -value** |
| DDD | -0.011 (-0.053, 0.03) | 0.5941 | 0.327 (-0.106, 0.761) | 0.1394 |
| Medical doctors | -0.134 (-0.246, -0.021) | **0.0202** | -0.029 (-0.404, 0.345) | 0.8775 |
| Gini index | -0.002 (-0.173, 0.169) | 0.9811 | -0.144 (-0.467, 0.18) | 0.3845 |
| C-section | 0.014 (0.003, 0.026) | **0.0132** | 0.033 (0.003, 0.063) | **0.0001** |
| Age | -0.143 (-0.156, -0.131) | **0.00004** | -0.224 (-0.278, -0.169) | **0.0319** |

95% CI: represents the 95% lower and upper bound confidence interval. DDD = defined daily dose, medical doctors = medical doctors per 10,000 population, and C-section = cesarean section rates per 1,000 population. The variables were standardised by subtracting their mean and dividing by their standard deviation (z-score standardisation) before fitting the model.


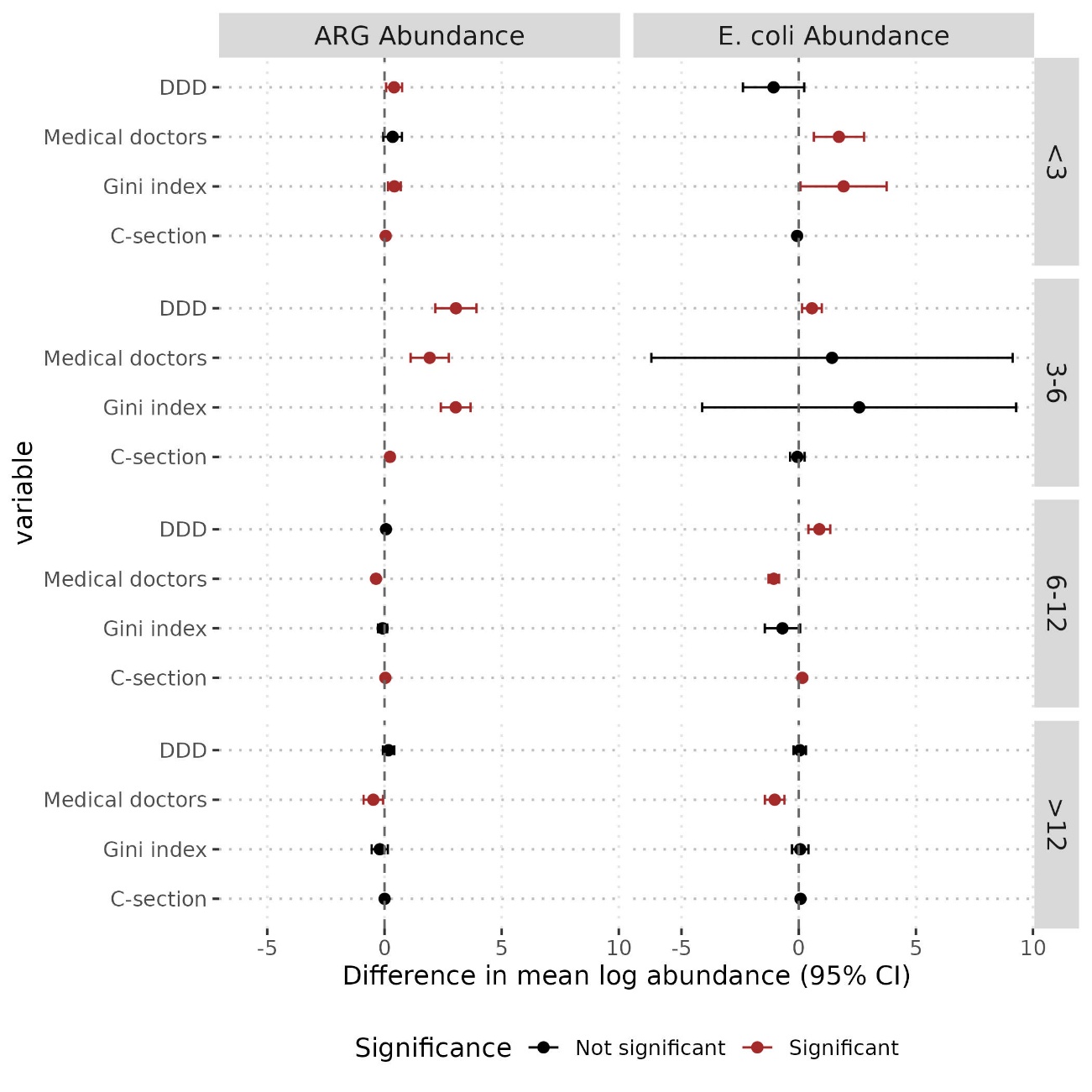


**Supplementary Fig. 13:** Association of national-level variables on resistome and *E. coli* abundance by age group. 95% CI: represents the 95% lower and upper bound confidence interval. DDD denotes defined daily dose of antibiotics consumed per 1,000 population; medical doctors refer to the number of medical doctors per 10, 000 population; C-section denotes percentage of caesarean section births; and age group is in months. The variables were standardised by subtracting their mean and dividing by their standard deviation (z-score standardisation) before fitting the model

**Supplementary Table 5**: Association of national-level variables on resistome and *E. coli* abundance by age group

| Age group | Variable | ARG abundance | | *E. coli* abundance | |
| --- | --- | --- | --- | --- | --- |
|  |  | **Difference in mean log ARG abundance (95% CI)** | ***P -* value** | **Difference in mean log *E. coli* abundance (95% CI)** | ***P* - value** |
| <3 | DDD | 0.409 (0.069, 0.750) | **0.0185** | -1.072 (-2.379, 0.234) | 0.1083 |
|  | Medical doctors | 0.348 (-0.048, 0.745) | 0.0852 | 1.717 (0.647, 2.787) | **0.0017** |
|  | Gini index | 0.421 (0.149, 0.693) | **0.0025** | 1.917 (0.079, 3.755) | **0.0414** |
|  | C-section | 0.052 (0.000, 0.103) | **0.0482** | -0.067 (-0.138, 0.003) | 0.0619 |
| 3-6 | DDD | 3.048 (2.169, 3.927) | **0.0000** | 0.565 (0.144, 0.985) | **0.0094** |
|  | Medical doctors | 1.929 (1.114, 2.744) | **0.0000** | 1.423 (-6.286, 9.132) | 0.7180 |
|  | Gini index | 3.037 (2.404, 3.671) | **0.0000** | 2.581 (-4.117, 9.280) | 0.4512 |
|  | C-section | 0.234 (0.098, 0.370) | **0.0009** | -0.059 (-0.372, 0.255) | 0.7147 |
| 6-12 | DDD | 0.064 (-0.005, 0.132) | 0.0697 | 0.881 (0.415, 1.346) | **0.0003** |
|  | Medical doctors | -0.363 (-0.486, -0.239) | **0.0000** | -1.064 (-1.291, -0.837) | **0.0001** |
|  | Gini index | -0.079 (-0.270, 0.112) | 0.4203 | -0.693 (-1.445, 0.059) | 0.0729 |
|  | C-section | 0.036 (0.023, 0.049) | **0.0000** | 0.155 (0.093, 0.217) | **0.0001** |
| >12 | DDD | 0.171 (-0.071, 0.414) | 0.1675 | 0.048 (-0.216, 0.311) | 0.7234 |
|  | Medical doctors | -0.478 (-0.893, -0.062) | **0.0251** | -1.024 (-1.441, -0.608) | **0.0001** |
|  | Gini index | -0.200 (-0.545, 0.144) | 0.2554 | 0.064 (-0.287, 0.416) | 0.7201 |
|  | C-section | 0.006 (-0.013, 0.024) | 0.5331 | 0.077 (-0.002, 0.156) | 0.0579 |

95% CI: represents the 95% lower and upper bound confidence interval. DDD denotes defined daily dose of antibiotics consumed per 1,000 population; medical doctors refer to the number of medical doctors per 10, 000 population; C-section denotes caesarean section births per 1,000 population; and age group is in months. The variables were standardised by subtracting their mean and dividing by their standard deviation (z-score standardisation) before fitting the model.
